# Yoda1 analogue (Dooku1) which antagonizes Yoda1‐evoked activation of Piezo1 and aortic relaxation

**DOI:** 10.1111/bph.14188

**Published:** 2018-04-06

**Authors:** Elizabeth L Evans, Kevin Cuthbertson, Naima Endesh, Baptiste Rode, Nicola M Blythe, Adam J Hyman, Sally J Hall, Hannah J Gaunt, Melanie J Ludlow, Richard Foster, David J Beech

**Affiliations:** ^1^ Leeds Institute of Cardiovascular and Metabolic Medicine, School of Medicine University of Leeds Leeds UK; ^2^ School of Chemistry University of Leeds Leeds UK

## Abstract

**Background and Purpose:**

The mechanosensitive Piezo1 channel has important roles in vascular physiology and disease. Yoda1 is a small‐molecule agonist, but the pharmacology of these channels is otherwise limited.

**Experimental Approach:**

Yoda1 analogues were generated by synthetic chemistry. Intracellular Ca^2+^ and Tl^+^ measurements were made in HEK 293 or CHO cell lines overexpressing channel subunits and in HUVECs, which natively express Piezo1. Isometric tension recordings were made from rings of mouse thoracic aorta.

**Key Results:**

Modification of the pyrazine ring of Yoda1 yielded an analogue, which lacked agonist activity but reversibly antagonized Yoda1. The analogue is referred to as Dooku1. Dooku1 inhibited 2 μM Yoda1‐induced Ca^2+^‐entry with IC_50_s of 1.3 μM (HEK 293 cells) and 1.5 μM (HUVECs) yet failed to inhibit constitutive Piezo1 channel activity. It had no effect on endogenous ATP‐evoked Ca^2+^ elevation or store‐operated Ca^2+^ entry in HEK 293 cells or Ca^2+^ entry through TRPV4 or TRPC4 channels overexpressed in CHO and HEK 293 cells. Yoda1 caused dose‐dependent relaxation of aortic rings, which was mediated by an endothelium‐ and NO‐dependent mechanism and which was antagonized by Dooku1 and analogues of Dooku1.

**Conclusion and Implications:**

Chemical antagonism of Yoda1‐evoked Piezo1 channel activity is possible, and the existence of a specific chemical interaction site is suggested with distinct binding and efficacy domains.

**Abbreviations**
SBS: standard bath solution
TRP: transient receptor potential

## Introduction

http://www.guidetopharmacology.org/GRAC/ObjectDisplayForward?objectId=2945 protein is important for mechanical force sensing and its transduction in higher organisms (Coste *et al.,* 2010; Ranade *et al.,* 2015; Wu *et al.,* 2016). It assembles as a trimer with a propeller‐like structure around a central ion pore, which is permeable to the cations Na^+^, K^+^ and Ca^2+^ (Coste *et al.,* 2012; 2015; Ge *et al.,* 2015; Guo and MacKinnon, 2017; Saotome *et al.,* 2017; Wu *et al.,* 2017; Zhao *et al.,* 2018). Mechanical forces that include membrane tension and laminar flow are able to activate the channel (Coste *et al.,* 2010; Li *et al.,* 2014; Lewis and Grandl, 2015; Syeda *et al.,* 2016).

Roles of Piezo1 have been identified in embryonic vascular maturation, BP regulation, physical performance, hypertension‐dependent arterial structural remodelling, urinary osmoregulation, epithelial homeostasis and axonal growth (Li *et al.,* 2014; Ranade *et al.,* 2014; Cahalan *et al.,* 2015; Retailleau *et al.,* 2015; Koser *et al.,* 2016; Martins *et al.,* 2016; Gudipaty *et al.,* 2017; Rode *et al.,* 2017). In addition, pathological significance of Piezo1 has been suggested in humans. Gain of function mutations have been linked to a form of haemolytic anaemia (hereditary stomatocytosis), and loss of function mutations have been linked to autosomal recessive congenital lymphatic dysplasia (Zarychanski *et al.,* 2012; Albuisson *et al.,* 2013; Andolfo *et al.,* 2013; Bae *et al.,* 2013; Fotiou *et al.,* 2015; Lukacs *et al.,* 2015).

Piezo1 pharmacology is in its infancy. Inhibitors of the channel are limited to generic inhibitors of the ion pore (http://www.guidetopharmacology.org/GRAC/LigandDisplayForward?ligandId=2426 and http://www.guidetopharmacology.org/GRAC/LigandDisplayForward?ligandId=2432) and the spider toxin http://www.guidetopharmacology.org/GRAC/LigandDisplayForward?ligandId=4206 which inhibits a range of mechanosensitive ion channels and may act indirectly *via* the lipid bilayer (Drew *et al.,* 2002; Suchyna *et al.,* 2004; Bowman *et al.,* 2007; Bae *et al.,* 2011). The first chemical activator of the channel, http://www.guidetopharmacology.org/GRAC/LigandDisplayForward?ligandId=9817, was discovered in 2015 through high‐throughput screening (Syeda *et al.,* 2015). Yoda1 is a useful research tool, not faithfully mimicking mechanical stimulation of the channels but facilitating study of Piezo1 channels from a practical perspective without the need for mechanical stimulation and importantly lacking effect on http://www.guidetopharmacology.org/GRAC/ObjectDisplayForward?objectId=2946 channels (Cahalan *et al.,* 2015; Lukacs *et al*., 2015; Wang *et al.,* 2016; Rode *et al.,* 2017).

As a step towards improved understanding of Piezo1 and the development of more and better Piezo1 modulators, increased knowledge on the structure–activity relationship for Yoda1 activation of Piezo1 would be helpful. Here, we addressed this knowledge gap by synthesizing Yoda1 analogues and testing them against the Piezo1 channel, other channels and vascular contractile function.

## Methods

### Piezo1 tetracycline‐inducible HEK 293 cell line

HEK T‐REx™ cells, which overexpress Piezo1 upon induction with tetracycline, were made as described in Rode *et al*. (2017). Expression was induced by treating the cells for 24 h with 10 ng·mL^−1^ tetracycline (Sigma) and analysed by quantitative RT‐PCR and Western blots.

### Cell culture

HEK 293 cells stably expressing tetracycline‐regulated human Piezo1 were utilized as described above. HEK 293 cells stably expressing tetracycline‐regulated human http://www.guidetopharmacology.org/GRAC/ObjectDisplayForward?objectId=489 have been described previously (Akbulut *et al.,* 2015). For the TRPC4‐expressing cells, selection was achieved by including 400 μg·mL^−1^ zeocin and 5 μg·mL^−1^ blasticidin in the cell medium. To induce expression, cells were incubated with 1 μg·mL^−1^ tetracycline for 24 h prior to experiments. All HEK 293 cells were maintained in DMEM (Invitrogen, Paisley, UK) supplemented with 10% FCS (Sigma‐Aldrich) and 1% penicillin/streptomycin (Sigma‐Aldrich).

HUVECs purchased from Lonza were maintained in Endothelial Cell Basal Medium. This media was supplemented with a bullet kit (Cell Media and Bullet Kit, Lonza) containing the growth factors: 10 ng·mL^−1^ VEGF, 5 ng·mL^−1^ human basic FGF, 1 μg·mL^−1^ hydrocortisone, 50 ng·mL^−1^ gentamicin, 50 ng·mL^−1^ amphotericin B and 10 μg·mL^−1^ heparin, in addition to 2% FCS (Sigma). HUVECs were passaged 2–6 times.

CHO K1 cells stably expressing human http://www.guidetopharmacology.org/GRAC/ObjectDisplayForward?objectId=510 were maintained in Ham's F12 (ThermoFisher Scientific) in the presence of 1 mg·mL^−1^ G418 (Sigma‐Aldrich).

All cells were grown at 37°C and in 5% CO_2_ in a humidified incubator.

### RT‐PCR

Total RNA was extracted using TRI reagent (Sigma‐Aldrich). Five hundred nanograms of total RNA was used for reverse transcription using the Reverse Transcription System (Promega). Real‐time PCR was conducted using an Applied Biosystems 7500 Real‐Time PCR system with intron spanning primers and Taqman probe for human Fam38A (Piezo1) (Hs00207230_m1) and GAPDH (Hs99999905_m1) (Applied Biosystems).

### Western blot for Piezo1 protein

Cells were harvested in lysis buffer [10 mM Tris (pH 7.4), 150 mM NaCl, 0.5 mM EDTA and 0.5% Nonidet P40 substitute] containing protease and phosphatase inhibitor cocktails (Sigma). Equal amounts of protein were loaded onto a 4–20% gradient gel (BioRad) and resolved by electrophoresis. Samples were transferred to PVDF membranes and labelled overnight with BEEC‐4 (1:1000, Cambridge Biosciences). HRP‐donkey anti‐rabbit secondary antibody (Jackson ImmunoResearch Laboratories) and SuperSignal Femto detection reagents (Pierce) were used for visualization.

### Intracellular Ca^2+^ measurements

HEK 293 and CHO cells were plated in poly‐d‐lysine coated 96‐well plates (Corning, NY, USA) and HUVECs in clear 96‐well plates (Corning, NY, USA) at a confluence of 90%, 24 h before experimentation. Cells were incubated with 2 μM fura‐2‐AM (Molecular Probes™) or 4 μM fluo‐4‐AM (for TRPV4 expressing CHO cells), in the presence of 0.01% pluronic acid (Thermo Fisher Scientific) in standard bath solution (SBS) for 1 h at 37°C. For recordings with fluo‐4, 2.5 mM probenecid (Sigma Aldrich) was included in the SBS throughout the experiment. Cells were washed with SBS for 30 min at room temperature. If inhibitors were being tested, these were added at this time, immediately following an SBS wash and maintained during the rest of the experiment. Measurements were made at room temperature on a 96‐well fluorescence plate reader (FlexStation, Molecular Devices, Sunnyvale, CA, USA) controlled by Softmax Pro software v5.4.5. For recordings using fura‐2, the change (Δ) in intracellular calcium was indicated as the ratio of fura‐2 emission (510 nm) intensities for 340 and 380 nm excitation. For recordings using fluo‐4, the dye was excited at 485 nm and emitted light collected at 525 nm, and measurements are shown as absolute fluorescence in arbitrary units. The SBS contained (mM): 130 NaCl, 5 KCl, 8 D‐glucose, 10 HEPES, 1.2 MgCl_2_, 1.5 CaCl_2_ and the pH was titrated to 7.4 with NaOH. For the Ca^2+^ add‐back experiments, Ca^2+^ free SBS was used (without CaCl_2_), and Ca^2+^ add‐back was 0.3 mM. For the washout experiments, inhibitors were washed 3 times with SBS immediately prior to recording.

### FluxOR™ intracellular Tl^+^ (thallium ion) measurements

Induced (Tet+) and non‐induced (Tet−) Piezo1 HEK 293 cells were plated in poly‐d‐lysine coated 96‐well plates (Corning, NY, USA) and HUVECs in clear 96‐well plates (Corning, NY, USA) at a confluence of 90%, 24 h before experimentation. Cells were loaded with FluxOR dye for 1 h at room temperature, before being transferred to assay buffer for 20 min. If inhibitors were being tested, these were added at this time and maintained throughout the experiment. Cells were stimulated with a Tl^+^‐containing K^+^‐free solution according to the manufacturer's instructions (Molecular Probes). Measurements were made at room temperature on a 96‐well fluorescence plate reader (FlexStation, Molecular Devices, Sunnyvale, CA, USA) controlled by Softmax Pro software v5.4.5. FluxOR was excited at 485 nm, emitted light collected at 520 nm, and measurements were expressed as a ratio increase over baseline (F/F_0_).

### Chemical synthesis of Yoda1 analogues

Analogues of Yoda1 were synthesized using three general synthetic approaches: 11 compounds [**2a‐2 k**] were synthesized using a one‐step procedure (Supporting Information Figure S1), compounds **7a** and **7b** using a four‐step procedure (Supporting Information Figure S2) and compound **11** using a separate four‐step procedure (Supporting Information Figure S3). All chemicals synthesized were purified by column chromatography or trituration and determined as >97% pure by ^1^H NMR (proton NMR) and ^13^C NMR (carbon‐13 NMR). Synthetic and analytical details are reported in the Supporting Information.

### Animals

Twelve to sixteen week‐old, wild‐type male C57BL/6 mice were used for experiments. All mice were housed in GM500 individually ventilated cages (Animal Care Systems) at 21°C, 50–70% humidity and with a 12 h alternating light/dark cycle. They had *ad libitum* access to RM1 diet (SpecialDiet Services, Witham, UK) with bedding from Pure'o Cell (Datesand, Manchester, UK). All animal experiments were authorized by the University of Leeds Animal Ethics Committee and the UK Home Office. Animal studies are reported in compliance with the ARRIVE guidelines (Kilkenny *et al.,* 2010; McGrath and Lilley, 2015).

### Aorta contraction studies

The wire myograph technique using vessels from mice is regarded as a useful model for studying vascular reactivity (Outzen *et al.,* 2015). Animals were killed by CO_2_ inhalation, according to Schedule 1 procedure approved by the UK Home Office. Thoracic aorta was dissected out and immediately placed into ice‐cold Krebs solution (125 mM NaCl, 3.8 mM KCl, 1.2 mM CaCl_2_, 25 mM NaHCO_3_, 1.2 mM KH_2_PO_4_, 1.5 mM MgSO_4_, 0.02 mM EDTA and 8 mM D‐glucose, pH 7.4). Connective tissue and fat were carefully removed under a dissection microscope. Segments, 1 mm long, were mounted in an isometric wire myograph system (Multi Wire Myograph System, 620 M, Danish Myo Technology) with two 40 μm diameter stainless steel wires, bathed in Krebs solution at 37°C and bubbled with 95% O_2_, 5% CO_2._ The segment was then stretched stepwise to its optimum resting tension to a 90% equivalent transmural pressure of 100 mmHg and equilibrated for 1 h prior to experiments. The stretch was approximately equal to that expected at diastolic BP (Rode *et al.,* 2017).

### Data and statistical analysis

The data and statistical analysis comply with the recommendations on experimental design and analysis in pharmacology (Curtis *et al.,* 2015). OriginPro 2015 (OriginLab, Northampton, MA, USA) was used for all data analysis. Averaged data are presented as mean ± SEM, where *n* represents the number of independent experiments for a given result and *N* indicates the total number of replicates within the independent experiments. Technical replicates were used to improve the confidence in data from independent experiments. In order to compare the pharmacological activity of Yoda1 analogues, data were normalized to the response of Yoda1 (agonist experiments) or the response of Yoda1 following pretreatment with vehicle only (inhibitor experiments). Data subjected to statistical analysis contained at least five independent experiments (*n*). For comparisons between two sets of data, Student's *t*‐tests were used. For multiple comparisons, one‐way ANOVA was used with Tukey's *post hoc* test. *P* < 0.05 was deemed significant. For IC_50_ determination, data were normalized to the vehicle controls (DMSO), and curves were fitted using the Hill1 (Origin Pro 2015) equation. The analogues were novel, and so, their initial testing occurred without knowledge of what effects might occur. Later in the study, analogues were blinded for aorta contraction experiments and used in random order. Randomization and blinding were not otherwise used.

### Materials

Unless stated otherwise, all commercially available chemicals were purchased from Sigma‐Aldrich. Stocks of chemicals were reconstituted in DMSO and stored at −20°C unless stated otherwise. Fura‐2‐AM and fluo‐4‐AM (Molecular Probes) were dissolved at 1 mM. Pluronic acid F‐127 was stored at 10% w^.^v^‐1^ in DMSO at room temperature. Probenecid was freshly prepared in 0.5 M NaOH and diluted 1:200 in SBS to give a working concentration of 2.5 mM. Yoda1 (Tocris) was stored at 10 mM. All Yoda1 analogues were synthesized and purified (for more information, see Supporting Information) and prepared as 10 mM stock solutions. Stock solutions were diluted 1:500 in the recording solution to give a final working concentration of 0.02% DMSO. http://www.guidetopharmacology.org/GRAC/LigandDisplayForward?ligandId=5351 and http://www.guidetopharmacology.org/GRAC/LigandDisplayForward?ligandId=2500 were stored as 5 and 10 mM stocks respectively. http://www.guidetopharmacology.org/GRAC/LigandDisplayForward?ligandId=8372 was prepared as a 10 mM stock solution and stored at −80°C. In experiments, (‐)‐Englerin A was used in SBS containing 0.01% pluronic acid as a dispersing agent to minimize aggregation of compound. http://www.guidetopharmacology.org/GRAC/LigandDisplayForward?ligandId=485 was stored at 100 mM in an aqueous solution. http://www.guidetopharmacology.org/GRAC/LigandDisplayForward?ligandId=1713 was stored at 10 mM in an aqueous stock solution. http://www.guidetopharmacology.org/GRAC/LigandDisplayForward?ligandId=1888 was stored as a 10 mM stock in water. SIN‐1 was stored as a 20 mM stock. BEEC‐4 anti‐Piezo1 antiserum, diluted 1:1000 for experiments, was generated by Cambridge Biosciences in rabbits by presentation of the Piezo1 peptide DLAKGGTVEYANEKHMLALA.

### Nomenclature of targets and ligands

Key protein targets and ligands in this article are hyperlinked to corresponding entries in http://www.guidetopharmacology.org, the common portal for data from the IUPHAR/BPS Guide to PHARMACOLOGY (Harding *et al.,* 2018), and are permanently archived in the Concise Guide to PHARMACOLOGY 2017/2018 (Alexander *et al.,* 2017a,b).

## Results

### 2,6‐Dichlorophenyl ring of Yoda1 is important for Piezo1 activity

**Figure 1:**
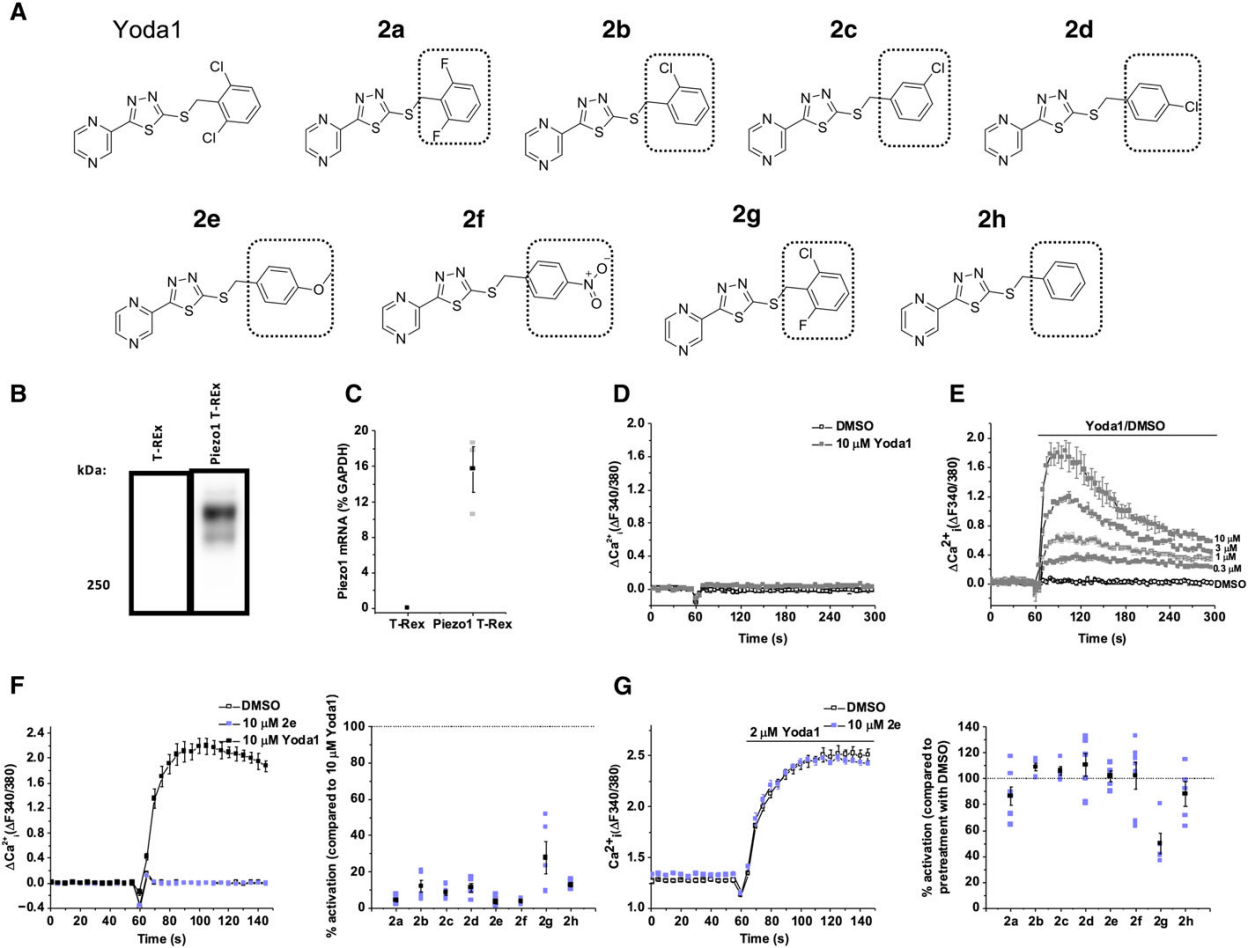
The 2,6‐dichlorophenyl group of Yoda1 is required for activation of Piezo1. (A) Structures of Yoda1 and analogues. Structural variation to Yoda1 is highlighted by the box outline. (B) Western blot of control T‐REx and Piezo1 T‐REx cells with anti‐Piezo1 antibody, confirming Piezo1 expression (predicted size, 286 kDa). (C) Real‐time PCR of Piezo1 mRNA levels relative to GAPDH mRNA in T‐REx and Piezo1 T‐REx cells. Error bars indicate SEM (*n* = 3). (D and E) FlexStation intracellular Ca^2+^ measurement data for T‐REx cells (D) and Piezo1 T‐REx cells (E) exposed to Yoda1 at the specified concentrations or exposed to the vehicle only (DMSO). (F) (Left) FlexStation intracellular Ca^2+^ measurement data for Piezo1 T‐REx cells exposed to 10 μM **2e** or exposed to vehicle only (DMSO). Error bars indicate SEM (*N* = 3). (Right) Summary for experiments of the type shown on the left measured between 40–60 s after Yoda1 analogue application, expressed as a % of the 10 μM Yoda1 response. Each data point represents a value from an independent experiment with mean values and error bars representing SEM indicated in black (*n* = 5). (G) (Left) FlexStation intracellular Ca^2+^ measurement data for Piezo1 T‐REx cells exposed to 2 μM Yoda1 after pretreatment with 10 μM **2e** or vehicle only (DMSO). Error bars indicate SEM (*N* = 3). (Right) Summary for experiments of the type shown on the left, as for (F, right) except data are expressed as a % of the Yoda1 response when pretreated with vehicle only (DMSO) (*n* = 5; **2b**, **2c**, **2e**, **2g**, **2h**, *n* = 7; **2a**, **2d**, **2f**).

We synthesized a series of Yoda1 analogues, focusing on simple modifications to the 2,6‐dichlorophenyl ring (Figure 1A). To reliably study the effects of Yoda1 analogues on overexpressed Piezo1 channels, we stably incorporated tetracycline‐inducible human Piezo1 expression in HEK 293 T‐REx™ cells. These cells, hereby referred to as Piezo1 T‐REx cells, showed Piezo1 expression after tetracycline induction but not without induction (Figure 1B, C) and displayed dose‐dependent Ca^2+^ entry in response to Yoda1, in comparison with normal HEK 293 T‐REx™ cells (without Piezo1 incorporation) that showed no response (Figure 1D, E). The Yoda1 analogues were screened at 10 μM for their ability to cause Ca^2+^ entry in these Piezo1 T‐REx cells and compared with the Ca^2+^ entry caused by the same concentration of Yoda1 (Figure 1F). All of the structural changes caused Piezo1 activation to be lost or mostly lost, with all compounds showing less than 30% activation compared with Yoda1 (Figure 1F).

The analogues were also screened for their ability to inhibit the Yoda1 response (Figure 1G). Each analogue was pre‐incubated with the cells for 30 min at 10 μM, prior to the application of 2 μM Yoda1 in the continued presence of the analogue. Pre‐incubation with these analogues did not affect the Ca^2+^ entry evoked by Yoda1, apart from **2g** which caused inhibition. These data suggest that the 2,6‐dichlorophenyl moiety of Yoda1 is essential for interacting with the Piezo1 channel. Only analogue **2g** had any effect, showing a slight inhibitory effect but little agonist effect; it is chemically similar to Yoda1 but with one fluorine replacing one chlorine.

### Identification of a Yoda1 analogue which antagonizes Yoda1

**Figure 2:**
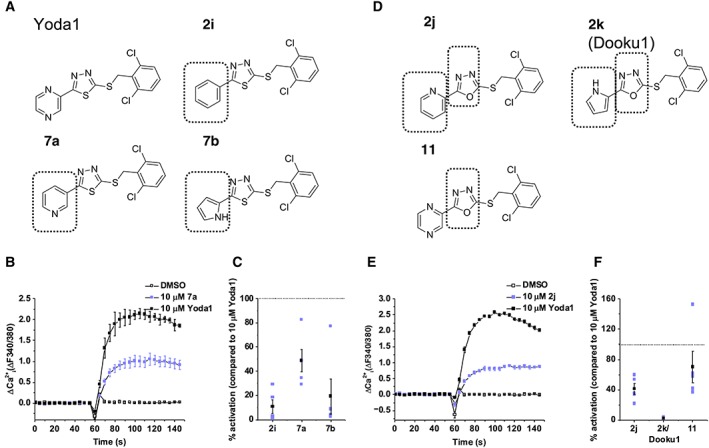
Changes to the pyrazine ring or replacing the thiadiazole with an oxadiazole give rise to less active analogues. (A) Structures of Yoda1 and analogues with changes to the pyrazine ring. Structural variation to Yoda1 is highlighted by the box outline. (B) FlexStation intracellular Ca^2+^ measurement data for Piezo1 T‐REx cells exposed to 10 μM **7a** or exposed to vehicle only (DMSO). Error bars indicate SEM (*N* = 3). (C) Summary for experiments of the type shown in (B) measured between 40–60 s after Yoda1 analogue application, expressed as a % of the 10 μM Yoda1 response. Each data point represents a value from an independent experiment with mean values and error bars representing SEM indicated in black (*n* = 5). (D) Structures of Yoda1 analogues with an oxadiazole. Structural variation to Yoda1 is highlighted by the box outline. (E) FlexStation intracellular Ca^2+^ measurement data for Piezo1 T‐REx cells exposed to 10 μM **2j** or exposed to vehicle only (DMSO). Error bars indicate SEM (*N* = 3). (F) Summary for experiments of the type shown in (E), as for (C).

To further investigate the structure–activity relationship of Yoda1, we synthesized analogues of the pyrazine group (Figure 2A). Similarly, these analogues were tested at 10 μM for their ability to cause Ca^2+^ entry in Piezo1 T‐REx cells, compared with Yoda1 (Figure 2B, C). Modification to the pyrazine ring significantly reduced activity in comparison with Yoda1, but analogue **7a** reached 50% of Yoda1 activity (Figure 2B, C). We then synthesized analogues of the thiadiazole group (Figure 2D) and tested these in the same manner (Figure 2E, F). Analogues containing an oxadiazole in place of a thiadiazole were also less active, but analogue **11**, the most similar in structure to Yoda1, showed ~70% activity (Figure 2E, F). These data suggest that the ability of Yoda1 to activate Piezo1 channels is dependent on very specific structural requirements but that changes to the pyrazine and thiadiazole groups can be tolerated.

**Figure 3:**
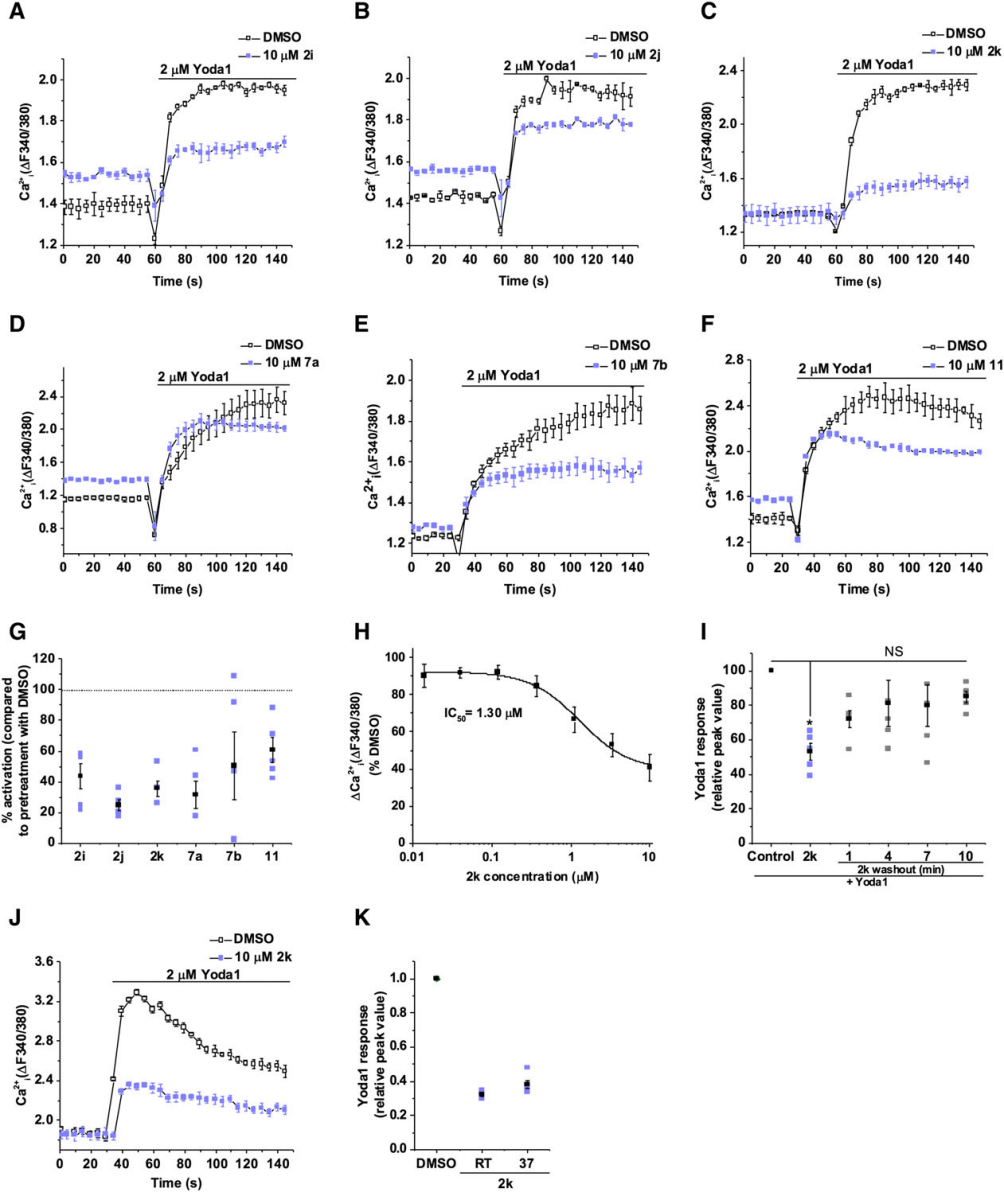
Yoda1 analogues are able to inhibit Yoda1‐induced Piezo1 activity. (A–F) FlexStation intracellular Ca^2+^ measurement data for Piezo1 T‐REx cells exposed to 2 μM Yoda1 after pretreatment with 10 μM **2i** (A), **2j** (B), **2k** (C), **7a** (D), **7b** (E), **11** (F) or vehicle only (DMSO). Error bars indicate SEM (*N* = 3). (G) Summary for experiments of the type shown in (A–F) measured between 40–60 s after Yoda1 analogue application, expressed as a % of the Yoda1 response when pretreated with vehicle only (DMSO). Each data point represents a value from an independent experiment with mean values and error bars representing SEM indicated in black (*n* = 5). (H) Mean data for the type of experiment shown in (C) with cells pretreated with indicated concentrations of **2k**. Expressed as a % of the Yoda1 response when pretreated with vehicle only (DMSO). The fitted curve is the Hill equation with IC_50_ 1.30 μM (*n* = 5). (I) Summary of intracellular Ca^2+^ measurement data (as for G) for Tet + Piezo1 T‐REx cells exposed to 2 μM Yoda1, following pretreatment with 10 μM **2k** or vehicle only (DMSO); **2k** was washed out before the recording (*n* = 5). (J) As for (C) but conducted at 37°C. (K) Summary for experiments of the type shown in (J) (*n* = 5).

To investigate whether these analogues could inhibit Yoda1 activity, we pre‐incubated cells with analogues and then tested Yoda1 (Figure 3A–G). The Yoda1 response was reduced by all analogues (Figure 3G). Analogues **2i** (Figure 3A), **2j** (Figure 3B), **7a** (Figure 3D), **7b** (Figure 3E) and **11** (Figure 3F) also had agonist activity, as shown by the elevated baseline Ca^2+^ signal compared with vehicle (DMSO) control. In contrast, analogue **2k** (Figure 3C) inhibited the Yoda1 response without changing the baseline and so lacked agonist activity (Figure 3C). Analogue **2k** was found to cause concentration‐dependent inhibition of Yoda1‐induced Ca^2+^ entry with an IC_50_ value of 1.30 μM (Figure 3H). Inhibition was incomplete at 10 μM, but higher concentrations of **2k** were not investigated because of solubility limitations. Recovery from the inhibitory effect of **2k** occurred after its washout (Figure 3I). The inhibitory effect of **2k** was not significantly different at 37°C compared with room temperature (Figure 3J, K). The data suggest that **2k** is an antagonist of Yoda1 that lacks agonist capability. We named **2k**, Dooku1.

### Dooku1 (analogue 2k) has selectivity for Piezo1

**Figure 4:**
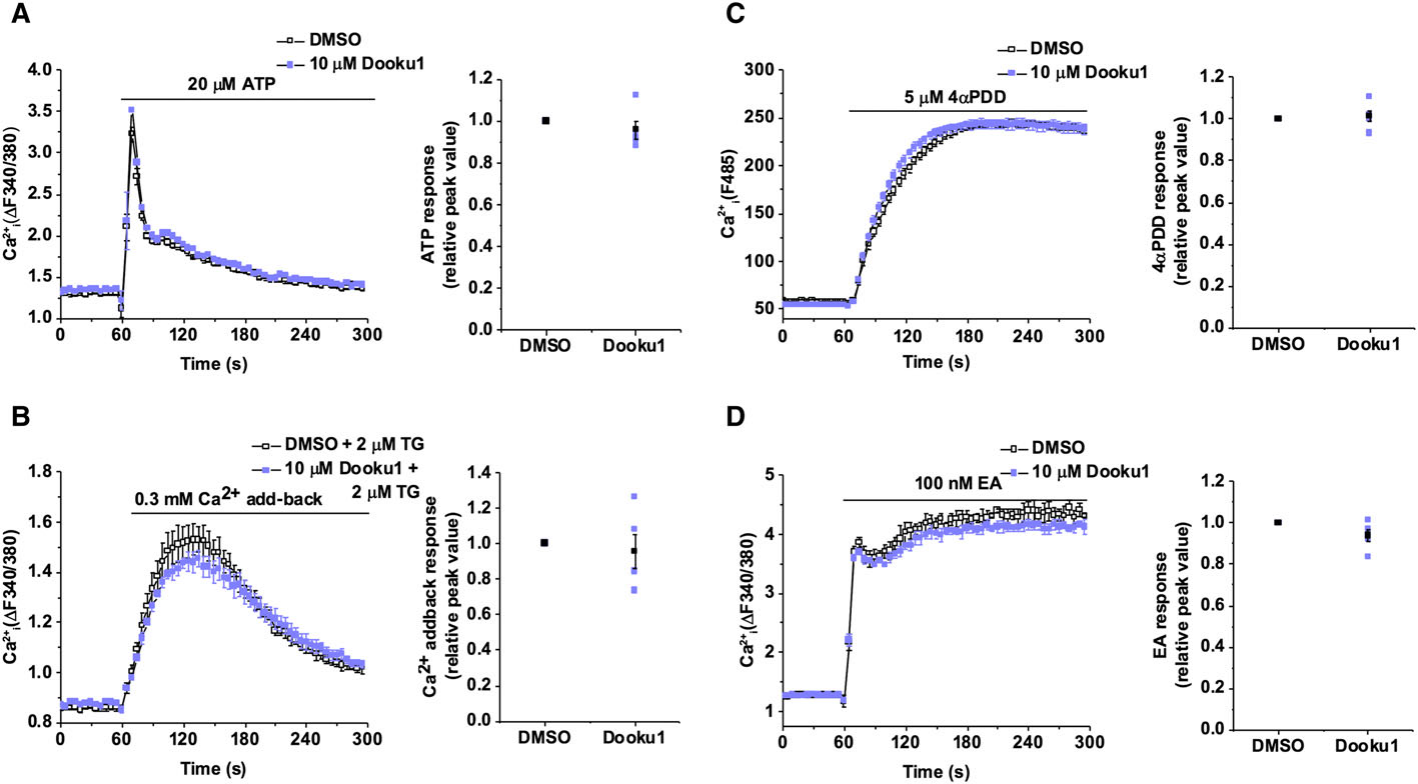
Selectivity of Dooku1. Ca^2+^ indicator dyes were fura‐2 (A, B, D) or fluo‐4 (C). Experiments conducted in native HEK 293 cells (A, B), CHO cells overexpressing TRPV4 (C) or HEK 293 cells overexpressing TRPC4 (D). Intracellular Ca^2+^ measurement data for cells exposed to 20 μM ATP (A), 0.3 mM Ca^2+^ addback (B), 5 μM 4α‐phorbol 12,13‐didecanoate (4α‐PDD) (C) or 100 nM (‐)‐Englerin A (EA) (D) following pretreatment with DMSO or 10 μM Dooku1 (left). Error bars indicate SEM (*N* = 3). Summary for experiments of the type shown on the left measured between 10–30 s (A), 60–90 s (B), 220–240 s (C) or 20–60 s (D) after treatment application and normalized to the peak amplitude values for the vehicle only (DMSO) pretreatment condition (right). Each data point represents a value from an independent experiment with mean values and error bars representing SEM indicated in black (*n* = 5).

Pretreatment with 10 μM Dooku1 had no effect on endogenous Ca^2+^ release in native HEK 293 cells in response to 20 μM ATP (Figure 4A). Dooku1 (10 μM) had no effect on store‐operated Ca^2+^ entry in HEK 293 cells: the Ca^2+^ addback response after intracellular Ca^2+^ store depletion by 2 μM thapsigargin (Figure 4B). Dooku1 (10 μM) had no effect on Ca^2+^ entry through TRPV4 channels overexpressed in CHO cells and activated by 4αPDD (Figure 4C) or on Ca^2+^ entry through TRPC4 channels overexpressed in T‐REx™ HEK 293 cells and activated by 100 nM (‐)‐Englerin A (EA) (Figure 4D). The data suggest selectivity of Dooku1 for Piezo1 channels.

### Dooku1 does not inhibit constitutive Piezo1 activity

**Figure 5:**
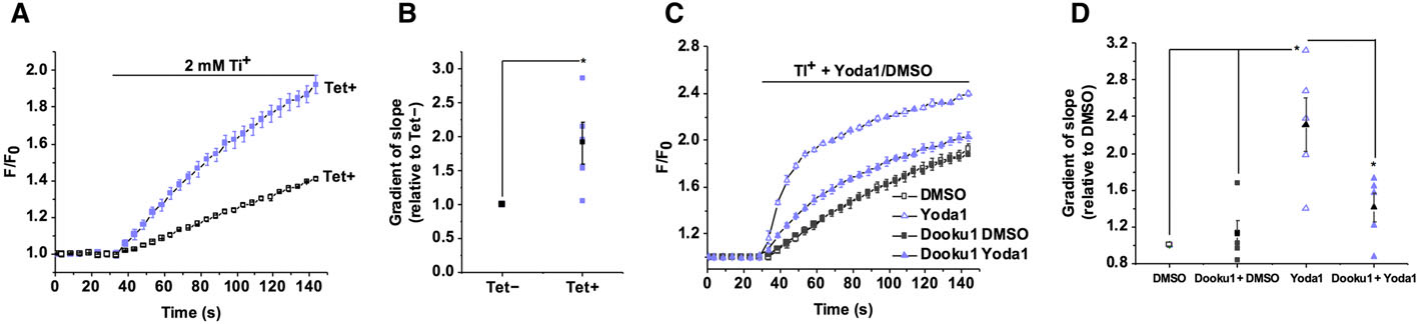
Dooku1 does not affect Piezo1 constitutive activity (A) Intracellular Tl^+^ measurement data using FluxOR for Tet + Piezo1 T‐REx cells or control Tet− cells exposed to extracellular Tl^+^. The FluxOR measurements are displayed as the fluorescence intensity (F) divided by the initial fluorescence intensity (F_0_). Error bars indicate SEM (*N* = 3). (B) Summary for experiments of the type shown in (A) measured between 0–30 s after Tl^+^ application, normalized to rate of change of F in the Tet− response. Each data point represents a value from an independent experiment with mean values and error bars representing SEM indicated in black (*n* = 5). (C) Intracellular Tl^+^ measurement data for Tet + Piezo1 T‐REx cells exposed to extracellular Tl^+^ and 5 μM Yoda1 or vehicle (DMSO), following pretreatment with 10 μM Dooku1 or vehicle only (DMSO). Error bars indicate SEM (*N* = 3). (D) Summary for experiments of the type shown in (C), as for (B) except data are normalized to the rate of change of the vehicle only (DMSO) control condition (*n* = 5).

To investigate whether the effect of Dooku1 depends on Yoda1, we took advantage of constitutive Piezo1 channel activity observed in the Piezo1 T‐REx cells (Rode *et al.,* 2017). The activity can be detected using an intracellular thallium (Tl^+^) sensitive FluxOR™ indicator dye whereby Tl^+^ influx acts as a surrogate for Na^+^ influx (Rode *et al.,* 2017). Cells were maintained in a Tl^+^ free solution until 2 μM Tl^+^ was added extracellularly 30 s into the recording, and the resulting elevation of intracellular Tl^+^ was detected. To ensure that constitutive Piezo1 channel activity was being represented in this assay, we compared the rate of Tl^+^ entry in tetracycline‐induced (Tet+) Piezo1 overexpressing cells to control cells to which tetracycline was not added (Tet−) (Figure 5A, B). The initial rate of Tl^+^ entry in the Tet + cells was nearly double that of control Tet− cells (Figure 5A, B).

Pretreatment with Dooku1 did not reduce constitutive Piezo1 channel activity as shown by comparing the DMSO and Dooku1 DMSO data (Figure 5C, D). Yoda1 increased the rate of Tl^+^ entry by ~2.5‐fold, and this effect was inhibited by 10 μM Dooku1 as shown by comparing the Yoda1 and Dooku1 Yoda1 data (Figure 5C, D). These data suggest that Dooku1 has no effect on constitutive Piezo1 channel activity and therefore that its effect depends on the presence of Yoda1.

### Dooku1 inhibits endogenous Yoda1‐activated channels

**Figure 6:**
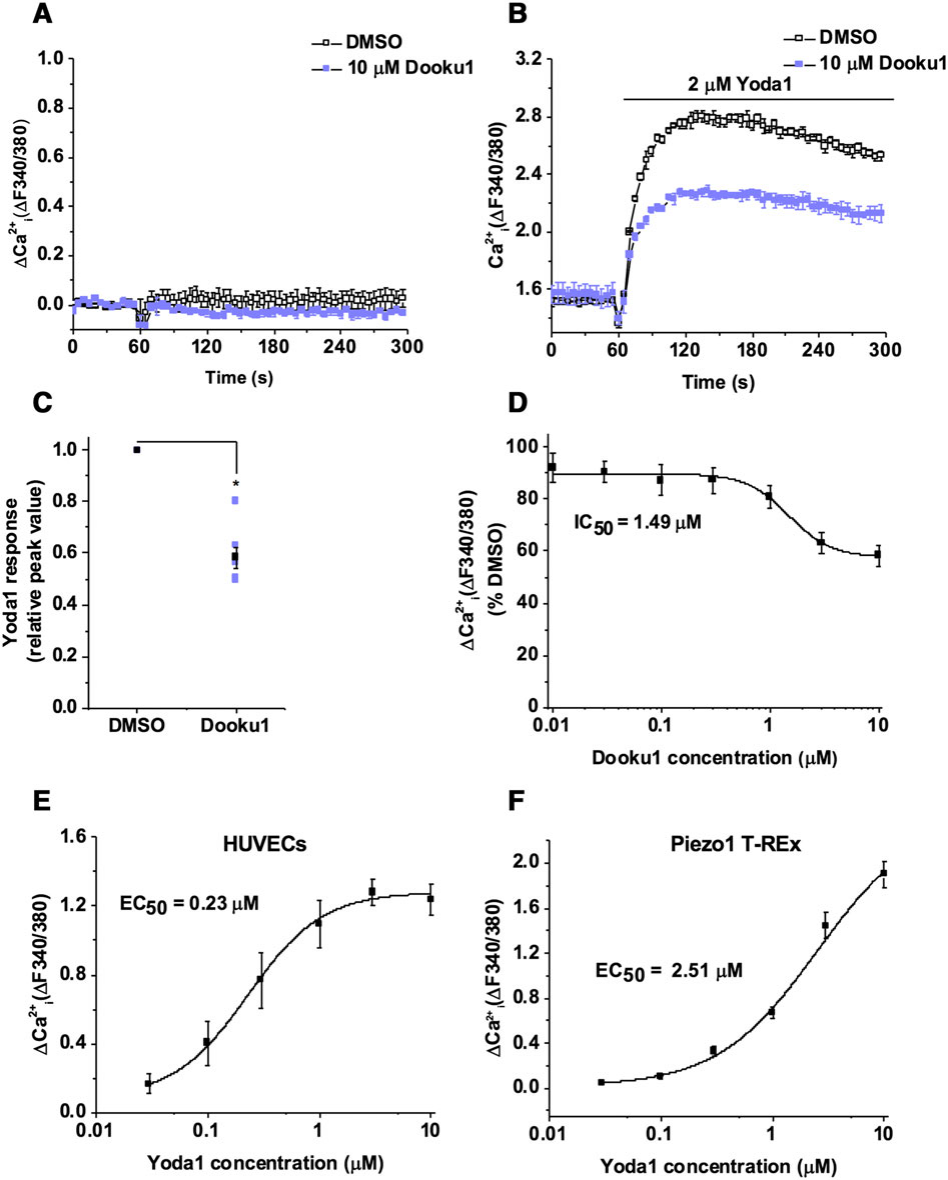
Dooku1 is effective against the endogenous Piezo1 channel. (A) Intracellular Ca^2+^ in HUVECs after exposure to 10 μM Dooku1 or vehicle only (DMSO). Error bars indicate SEM (*N* = 3). (B) Intracellular Ca^2+^ in HUVECs after exposure to 2 μM Yoda1 after pretreatment with 10 μM Dooku1 or vehicle only (DMSO). Error bars indicate SEM (*N* = 3). (C) Summary for experiments of the type shown in (B) measured 40–60 s after Yoda1 application and normalized to peak amplitudes for the vehicle only group. Each data point represents a value from an independent experiment with mean values and error bars representing SEM indicated in black (*n* = 7). (D) Mean data for the type of experiment shown in (B) with cells pretreated with indicated concentrations of Dooku1. Data are expressed as a % of the Yoda1 response when pretreated with vehicle only (DMSO). The fitted curve is the Hill equation with IC_50_ 1.49 μM (*n* = 5). (E, F) Mean intracellular Ca^2+^ for HUVECs (E) or Piezo1 T‐REx cells (F) exposed to the indicated concentrations of Yoda1. The fitted curve is the Hill equation with EC_50_ of 0.23 μM (E) and 2.51 μM (F) (*n* = 3).

The above studies were on overexpressed Piezo1 channels. To investigate the relevance to endogenous Piezo1 channels, we studied HUVECs that robustly express endogenous Piezo1 channels (Li *et al.,* 2014) and display a Piezo1‐dependent Yoda1 response (Rode *et al.,* 2017). Similar to observations in Piezo1 T‐REx cells (Figure 3C), Dooku1 did not evoke Ca^2+^ entry (Figure 6A). Dooku1 was however able to inhibit the Yoda1 response in HUVECs (Figures 6B, C). Dooku1 had a concentration‐dependent inhibitory effect against Yoda1‐induced Ca^2+^ entry in HUVECs, acting with an IC_50_ of 1.49 μM (Figure 6D), which was comparable with the value in Piezo1 T‐REx cells even though its maximum effect was less (Figure 3H). These data suggest that Dooku1 is also an antagonist of Yoda1‐induced Piezo1 channels in endothelial cells. To investigate the reason for reduced Dooku1 effect against the endogenous Yoda1‐activated channel, we compared the concentration‐effect curves of Yoda1 in HUVECs (Figure 6E) and Piezo1 T‐REx cells (Figure 6F). Yoda1 had increased potency in HUVECs with an EC_50_ of 0.23 μM, compared with 2.51 μM in Piezo1 T‐REx cells, suggesting that greater Yoda1 potency in HUVECs may explain the smaller effect of Dooku1 in HUVECs.

### Yoda1 causes endothelium‐dependent and NO‐dependent relaxation of aorta

**Figure 7:**
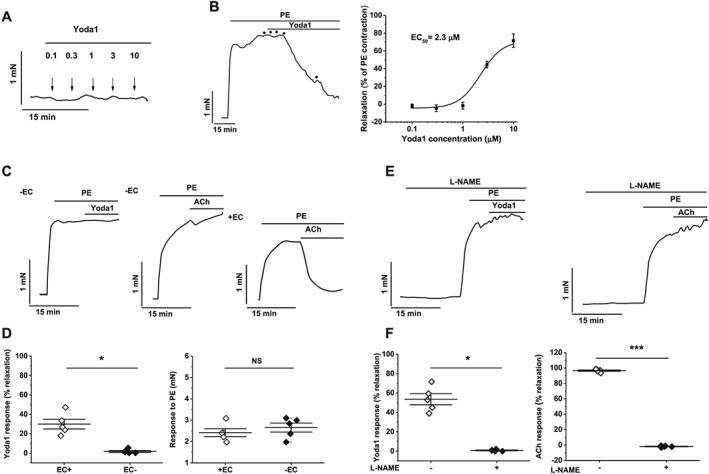
Yoda1‐induced relaxation in mouse thoracic aorta is endothelium‐ and NO‐dependent. (A) Isometric tension recording from aorta exposed to the indicated concentrations of Yoda1. (B) (left) As for (A) but following pre‐constriction with 0.3 μM phenylephrine (PE). (Right) Mean data for experiments of the type shown on the left expressed as % relaxation. The fitted curve is the Hill equation with EC_50_ of 2.3 μM (*n* = 5). (C) Isometric tension recording of aorta pre‐constricted with PE and exposed to 5 μM Yoda1 (left) or 5 μM ACh control (middle and right) with the endothelial layer removed (left and middle) or intact (right). (D) Summary data for experiments of the type shown in (B and C, left) expressed as % relaxation evoked by Yoda1 (left) or the response to PE (right) in the presence (EC+) or absence (EC−) of the endothelial cell layer. Each data point represents a value from an independent experiment with mean values and error bars representing SEM indicated by the black lines (*n* = 5). (E) As for (C) but following pre‐incubation with 100 μM N^ω‐^nitro‐L‐arginine methyl ester (L‐NAME). (F) As for (D) but for experiments of the type shown in (E).

To investigate physiological responses, we made isometric tension recordings from isolated murine thoracic aorta rings. Yoda1 had no effect in the absence of phenylephrine (PE), which is an agonist of α_1_‐adrenoreceptors (Figure 7A). Rings contracted in response to PE (Figure 7B) and Yoda1 caused concentration‐dependent relaxation following this pre‐contraction, with an estimated EC_50_ of 2.3 μM (Figure 7B). Endothelium‐denudation abolished the Yoda1 response but did not affect the PE response (Figure 7C, D). Response to ACh was a positive control for functional endothelium, and this response was present in endothelium‐intact rings but absent in endothelium‐denuded rings (Figure 7C). The data suggest that Yoda1 causes endothelium‐dependent relaxation in mouse thoracic aorta.

To determine whether the relaxation caused by Yoda1 was dependent on NOS, we exposed rings to NOS inhibitor, Nω‐nitro‐L‐arginine methyl ester (L‐NAME). L‐NAME prevented Yoda1‐induced and ACh‐induced relaxation (Figure 7E, F). The data suggest that Yoda1 causes endothelium‐dependent relaxation in mouse thoracic aorta by stimulating NO production *via* the endothelium.

### Dooku1 inhibits Yoda1‐induced relaxation of aorta

**Figure 8:**
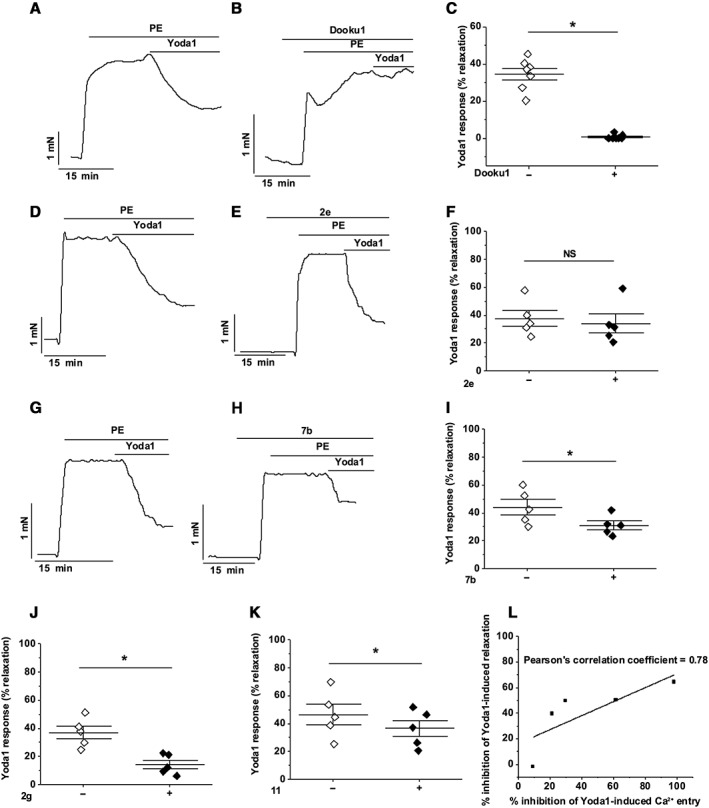
Dooku1 inhibits Yoda1‐induced dilation in aorta. (A–K) Isometric tension data from mouse thoracic aorta with intact endothelium. (A) Pre‐constricted with PE and exposed to 5 μM Yoda1. (B) As for (A) but following 30 min pre‐incubation with 10 μM Dooku1. (C) Summary data for experiments of the type shown in (A, B) expressed as % relaxation evoked by Yoda1. Each data point represents a value from an independent experiment with mean values and error bars representing SEM indicated by the black lines (*n* = 7). (D–F) (G–I) As for (A–C) but following pre‐incubation with 10 μM **2e** (D–F) or **7b** (G–I) (*n* = 5 on F, I). (J, K) As for (C) but following pre‐incubation with 10 μM **2g** (J) or **11** (K) (*n* = 5). (L) Comparison of the mean % inhibition of Yoda1‐induced relaxation in mouse thoracic aorta and the mean % inhibition of Yoda1‐induced Ca^2+^ entry by the five compounds: **2e**, **2g**, **Dooku1**, **7b** and **11**. The points are fit to a straight line with Pearson's correlation coefficient of 0.78.

To determine if Dooku1 inhibits relaxation caused by Yoda1, aortic rings were pre‐incubated with 10 μM Dooku1 for 20 min. Dooku1 strongly suppressed the Yoda1‐induced relaxation (Figure 8A–C). To characterize this phenomenon in more detail, we tested four further Yoda1 analogues in the aorta assay. The selected analogues showed various abilities to inhibit Yoda1 responses in Piezo1 T‐REx cells: analogues **2e** (no activation and no inhibition) (Figure 1), **2g** (slight activation and slight inhibition) (Figure 1), **7b** (slight activation and partial inhibition) (Figures 2 and 3) and **11** (slight activation and partial inhibition) (Figures 2 and 3). Analogue **2e** had no effect (Figure 8D–F). **2g**, **7b** and **11** in contrast suppressed the Yoda1‐induced relaxation (Figure 8G–K). Moreover, the ability of these analogues to inhibit Yoda1‐induced relaxation correlated with inhibition of Yoda1‐induced Ca^2+^ entry (Figure 8L). The data suggest strong efficacy of Dooku1 as an inhibitor of Yoda1‐induced aortic relaxation that is mediated through disruption of Yoda1‐induced Piezo1 channel activity.

### Dooku1 is selective for Yoda1‐induced relaxation but partially inhibits agonist contractile responses

**Figure 9:**
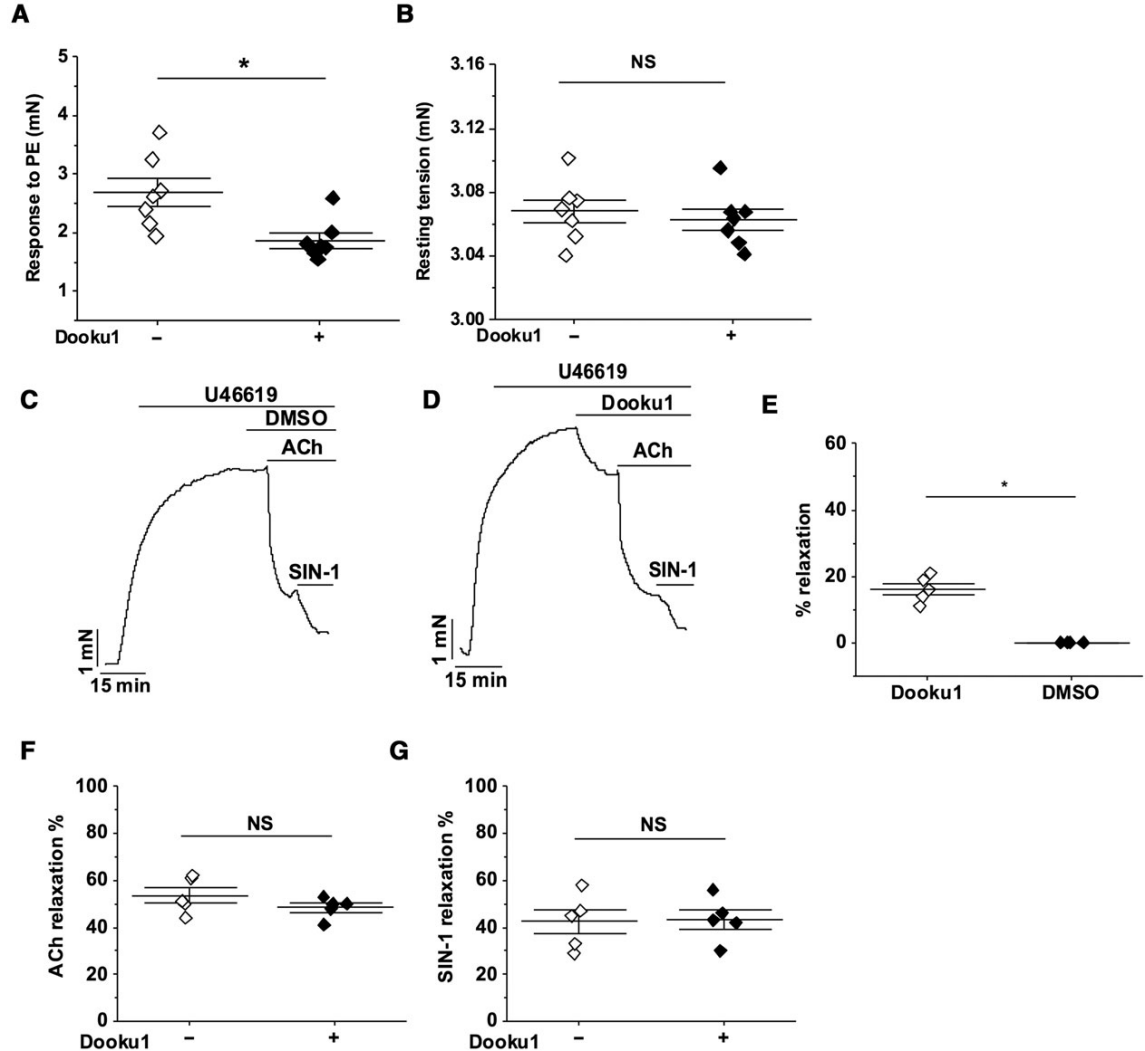
Specificity of Dooku1 in aorta. All experiments were performed on mouse thoracic aorta with intact endothelium. (A, B) Summary data for experiments of the type shown in Figure 8A, B, expressed as the response to PE (A) or resting tension (B) before and after pre‐incubation with 10 μM Dooku1. Each data point represents a value from an independent experiment with mean values and error bars representing SEM indicated by the black lines (*n* = 7). (C) Aorta were pre‐constricted with 0.1 μM U46619 and treated consecutively with DMSO, 1 μM ACh and 10 μM SIN‐1. (D) As for C but pretreated with Dooku1 instead of DMSO. (E–G) Summary data for experiments of the type shown in (C, D) expressed as % of the effect of Dooku1 on the contraction by U46619 (E) or % relaxation evoked by ACh (F) or SIN‐1 (G) before and after pre‐incubation with 10 μM Dooku1. Each data point represents a value from an independent experiment with mean values and error bars representing SEM indicated by the black lines (*n* = 5).

**Figure 10:**
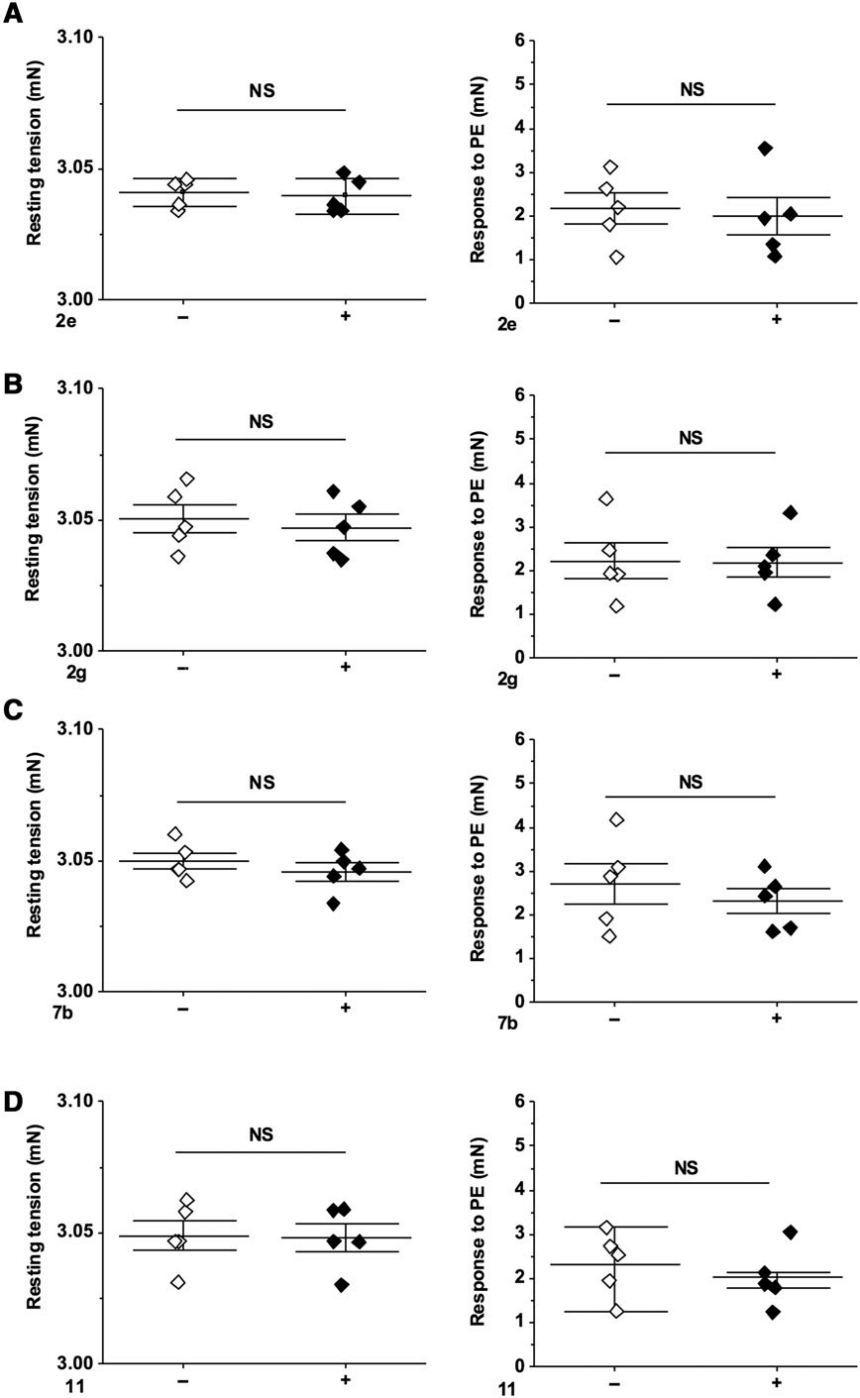
Lack of effect of other Yoda1 analogues on PE‐induced contraction. Summary data for experiments of the type shown in Figure 8 D–E, G–H expressed as resting tension (left) or the response to PE (right) following pre‐incubation with 10 μM **2e** (A), **2g** (B), **7b** (C) and **11** (D). Each data point represents a value from an independent experiment with mean values and error bars representing SEM indicated by the black lines (*n* = 5).

Analysis of the PE response in the presence of Dooku1 revealed significant inhibition without effect on baseline tension (Figure 9A, B). To determine whether Dooku1's inhibition of PE‐induced contraction was specific to this contractile agent, we also tested the effect of Dooku1 against contraction induced by U46619, a Tx A_2_ mimetic. Aortic rings were pre‐contracted with 0.1 μM U46619 (Figure 9C, D). Addition of Dooku1 caused partial relaxation (Figure 9D, E). In contrast, Dooku1 had no effect on relaxation evoked by ACh (1 μM) or the NO donor SIN‐1 (10 μM) (Figure 9F, G). Investigation of the PE response in the presence of the other four Yoda1 analogues revealed no inhibitory effect (Figure 10). The data suggest that Dooku1 selectively inhibits Yoda1‐induced relaxation but also partially inhibits receptor‐mediated agonist responses *via* unknown mechanisms.

## Discussion and conclusions

This study has provided insight into the structure–activity relationships for Piezo1 channel activation by Yoda1 with the goal of generating new tools for investigating Piezo1 channel function. Through this research, we have identified and named Dooku1, an inhibitor of Yoda1‐induced Piezo1 channel activity that strongly inhibits Yoda1‐induced relaxation of aorta. The data suggest that Dooku1 may compete with Yoda1 at a binding site or act allosterically at another site to reduce the binding or efficacy of Yoda1.

During the discovery of Yoda1, the 2,6‐dichlorophenyl group of the compound was highlighted as important with particular reference to the chlorine atoms (Syeda *et al.,* 2015). Our findings support this conclusion and add new knowledge by demonstrating that small changes to this group result in complete loss of Piezo1 channel activation. Removing one of the chlorine atoms [**2b**] or altering the position of the chlorine atom around the ring [**2c/2d**] abolished activity. Replacing one or both of the chlorine atoms with fluorine [**2a/g**] also abolished activity implying that both chlorine atoms are important for activity and may interact with Piezo1 in a chlorine specific manner, potentially *via* a σ‐hole interaction, such as a halogen‐pi bond. The 4‐methoxyphenyl [**2e**] and 4‐nitrophenyl [**2f**] analogues were also inactive. Investigating the inhibitory potential of the compounds showed that all but **2g**, which is the most similar in structure to Yoda1, were ineffective at inhibiting Yoda1 activity.

Piezo1 channel activation by analogues with modification to the pyrazine group was less than that of Yoda1, with the most successful analogue, compound **7a**, in which the pyrazine was replaced with a 3‐pyridyl group, exhibiting 50% of the activity of Yoda1. This demonstrates the importance of the nitrogen atom in the 2‐position of the pyrazine ring, with loss of this nitrogen resulting in a 50% drop of activity. The remaining two compounds from the series, the phenyl [**2i**] and 2‐pyrrolyl [**7b**] analogues, were less active than **7a** that suggests that the presence of the nitrogen atom at the 3‐position of the pyridine ring in **7a** is also contributing to Piezo1 activation, supporting our understanding of the importance of the nitrogen atom at the equivalent position on the pyrazine ring of Yoda1 to activity. We next investigated replacement of the central thiadiazole ring by an oxadiazole [**11**]. This change was largely tolerated with the new compound demonstrating 70% of the activity of Yoda1. The other two compounds from the series were less active, although the data for the 2‐pyridyl analogue [**2j]** were interesting in that the partial activity observed for the analogue suggests that the position of the nitrogen atom on the pyridine contributes to activity, reinforcing the importance of the equivalent *N* on the pyrazine ring of Yoda1 to activity.

Investigation into the inhibitory potential of this set of left‐hand and middle ring‐modified analogues provided compounds with potential promise of being pharmacological tools. All of the compounds from the series had the ability to reduce Ca^2+^ entry evoked by Yoda1 by at least 40%, and as much as 75% in the case of **2j**. However, most of these compounds exhibited partial agonist activity. The most promising compound, **2k** (Dooku1) effectively reduced Yoda1 activity by 60%, without causing any activation and was a strong inhibitor of the Yoda1 response in the physiological setting of murine aortic rings. This shows that the pyrazine ring can be replaced to identify compounds, which do not activate the channel but do inhibit the Yoda1 response. It appears that analogues lacking the 2,6‐dichlorophenyl group do not activate the channels or inhibit Yoda1 whereas pyrazine‐modified analogues show reduced activation and ability to inhibit Yoda1. Therefore, the di‐chloro group seems to be critical for binding while the pyrazine group is less important for binding but key for channel activation.

Currently, the only available inhibitors of Piezo1 activity are not selective for Piezo1 (Drew *et al.,* 2002; Bae *et al.,* 2011). Dooku1 is also not perfect as it does not directly block the channels, but it is a new tool compound that is useful for Piezo1 characterization studies. It antagonizes the action of Yoda1 and could facilitate understanding of an important small‐molecule binding site on or near to Piezo1 channels. Without agonist activity, Dooku1 effectively inhibits Yoda1‐induced Piezo1 activity. It does so without disturbing several Ca^2+^ handling events in the cell or affecting other aortic relaxing agents. Although these data suggest specificity of Dooku1 for Piezo1 channels, further studies to address this point are warranted, especially given the inhibitory effect of Dooku1 against PE and U46619‐induced contractions of aortic rings that might reflect a Piezo1 mechanism or some other unknown effect of Dooku1. It is possible that Dooku1 may be acting on Piezo1 in smooth muscle cells of the vessel, partially inhibiting contraction. This assumes that the channels become activated *via* a Yoda1‐like mechanism during contraction. Piezo1 was found not be required for normal myogenic tone (Retailleau *et al*., 2015), and so, a non‐Piezo1 target of Dooku1 should be considered.

Dooku1 only has activity against Yoda1‐induced and not constitutive Piezo1 channel activity. Such an effect is consistent with Dooku1 acting at the same or a similar site to Yoda1 and thereby occluding access of Yoda1 to its agonist binding site. The reversibility of Dooku1 is consistent with the reversibility of Yoda1 (Rocio Servin‐Vences *et al.,* 2017). It would be good to investigate if the Dooku1 effect is consistent with competitive antagonism, but solubility limitations of the compounds prevented construction of appropriate concentration–response curves. The inability of Dooku1 to have any effect on constitutive activity suggests that the mechanism of background channel activity is different to that of chemical activation with Yoda1.

Dooku1 partially inhibited Yoda1 in HUVECs but strongly inhibited it in aorta (Figure 6D cf. Figure 8C). We initially speculated that the difference was due to the higher temperature of the contraction studies (37°C cf. room temperature), but the Dooku1 effect was not significantly temperature dependent (Figure 3K). An alternative explanation might be that Ca^2+^ entry is not directly proportional to NO production, so that partial inhibition of Yoda‐1 induced Ca^2+^ entry is sufficient to inhibit most of the relaxation induced by Yoda1. Another divergence was that Yoda1 was more potent in HUVECs than Piezo1 T‐REx cells, showing a difference between native and over‐expressed Piezo1 channels (Figure 6E, F). We speculate that this difference reflected a higher basal state of activity of the channels in endothelial cells, as described previously (Rode *et al.,* 2017), making the channels more sensitive to Yoda1 because they are better primed for opening.

In summary, this study has provided important insight into the structure–activity relationships of Yoda1 and supported the concept of a specific chemical binding site on or in close proximity to Piezo1 channels. It has also revealed the discovery of a useful tool compound, Dooku1, which effectively antagonizes Yoda1‐induced Piezo1 channel activity, distinguishing it from constitutive Piezo1 channel activity. The complete role of Piezo1 in vascular biology is still being established, but the protein may have significant clinical interest with emerging roles in genetic disease, BP control, hypertension‐induced arterial remodelling and exercise capacity (Retailleau *et al.,* 2015; Wang *et al.,* 2016; Rode *et al.,* 2017). As yet, it is not clear whether activating or inhibiting this channel may be advantageous, but increasing our pharmacological knowledge, alongside our physiological knowledge of Piezo1 will be essential if therapeutic potential of this protein is to be harnessed in the future. Learning more about Piezo1 channel interactions with small‐molecules promises to be an important aspect of the overall effort to understand Piezo1 biology.

## Author contributions

E.L.E., K.C., N.E., B.R., N.M.B., A.J.H., S.J.H., H.J.G. and M.J.L. performed experiments and data analysis. E.L.E., K.C., D.J.B. and R.F. designed the research. D.J.B. and R.F. raised funds to support the work. E.L.E., K.C., R.F. and D.J.B. co‐wrote the paper.

## Conflict of interest

The authors declare no conflicts of interest.

## Declaration of transparency and scientific rigour

This http://onlinelibrary.wiley.com/doi/10.1111/bph.13405/abstract acknowledges that this paper adheres to the principles for transparent reporting and scientific rigour of preclinical research recommended by funding agencies, publishers and other organisations engaged with supporting research.

## Supporting information


**Figure S1** The general thiol alkylation reaction used to produce 11 compounds.
**Figure S2** General synthetic route towards 7a and 7b. 2,6‐dichlorobenzyl chloride (3) is first converted to the thiol 4 followed by an SNAr to give 5, which is then brominated to give 6 ready for a Suzuki cross‐coupling to give the desired products 7a‐b.
**Figure S3** Synthetic route for 10. 2,6‐dichlorobenzyl chloride (3) is first converted to the thiol 4 ready for a reaction with CDI and hydrazine to afford 8. Compound 8 is then utilized in an amide coupling with 9 using EDCI to produce 10 ready for a cyclising‐condensation reaction to afford 11.
