# Supplementary material for: Yoda1 analogue (Dooku1) which antagonizes Yoda1‐evoked activation of Piezo1 and aortic relaxation
Source: Br J Pharmacol. 2018 Apr 6;175(10):1744–59. doi: 10.1111/bph.14188 (PMC5913400; doi:10.1111/bph.14188)
Supplement: Supplementary file 1 — Figure S1 The general thiol alkylation reaction used to produce 11 compounds. Figure S2 General synthetic route towards 7a and 7b. 2,6‐dichlorobenzyl chloride (3) is first converted to the thiol 4 followed by an SNAr to give 5, which is then brominated to give 6 ready for a Suzuki cross‐coupling to give the desired products 7a‐b. Figure S3 Synthetic route for 10. 2,6‐dichlorobenzyl chloride (3) is first converted to the thiol 4 ready for a reaction with CDI and hydrazine to afford 8. Compound 8 is then utilized in an amide coupling with 9 using EDCI to produce 10 ready for a cyclising‐condensation reaction to afford 11. [file BPH-175-1744-s001.pdf]

SUPPLEMENTAL DATA

Materials

All purchased chemicals and solvents were used without further purification unless otherwise stated.

Physical Methods

^{1}H Nuclear Magnetic Resonance spectra were recorded at 500 MHz using a Bruker DRX 500 instrument or at 400 MHz using a Bruker DPX 400. ^{1}H spectra are referenced based on the residual proton in the solvent (*e.g.* the $CHCl_3$, 0.01 % in 99.99 % $CDCl_3$). Coupling constants (J) are reported to the nearest 0.1 Hz. ^{13}C NMR spectra were recorded at 125 MHz on 500 MHz spectrometers or at 100 MHz on 400 MHz spectrometers. Infrared spectra were recorded on a Bruker Alpha Platinum ATR FTIR spectrometer and samples analysed in the solid phase. LC-MS was performed on a Bruker Daltronics running a gradient of increasing acetonitrile (5 to 95 %) in water both containing 0.1 % formic acid at 1 ml/min, on a short path c18 reverse phase column, detecting compounds with both a diode array detector and a Bruker mass spectrum analyser. Biotage silica column chromatography was carried out using an Isolera Four EXP with Spektra. HPLC was performed on an Agilent 1290 Infinity Series equipped with a UV detector and Hyperprep C_{18} reverse phase column.

Key to NMR abbreviations

s (singlet), br s (broad singlet) d (doublet), dd (doublet of doublets), ddd (doublet of doublets of doublets), t (triplet), dt (doublet of triples), q (quartet), m (multiplet).

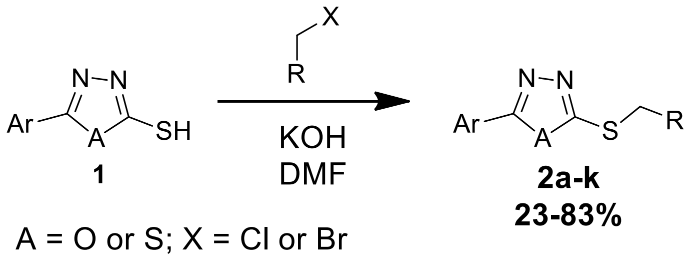

Figure S1: The general thiol alkylation reaction used to produce 11 compounds.

General Procedure A

An aromatic thiol (0.28 mmol, 1 eq) and KOH (0.34 mmol, 1.2 eq) were added to degassed DMF (5 mL) under a flow of nitrogen. This solution was stirred for 1 h followed by dropwise addition of a benzyl halide (0.31 mmol, 1.1 eq) in degassed DMF (2 mL) over 25 minutes, solution heated to 80 ^{0}C

and allowed to stir for 2 h. The reaction mix was then diluted with H_2O (20 mL) and extracted with EtOAc (3 x 15 mL), the organic layers were combined and washed with sat. NH_4Cl solution (2 x 15 mL), sat. $NaHCO_3$ solution (2 x 15 mL), sat. NaCl solution (2 x 15 mL), 10% LiCl solution (w/w) (2 x 15 mL), dried over Na_2SO_4, filtered and evaporated to dryness *in vacuo* to afford a crude product.

2-((2,6-difluorobenzyl)thio)-5-(pyrazin-2-yl)-1,3,4-thiadiazole (2a)

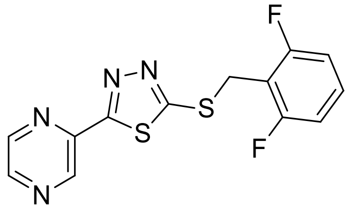

General procedure A was followed using 5-(2-pyrazinyl)-1,3,4-thiadiazole-2-thiol (35 mg, 0.18 mmol), KOH (12 mg, 0.21 mmol) and 2,6-dichlorofluorobenzyl bromide (41 mg, 0.20 mmol) to afford a pale yellow solid (56 mg). This was purified by ACC (0-60% EtOAc in Petrol) to afford yellow needles (28 mg, 0.09 mmol, 50%). Rf 0.45 (7:3 Petrol:EtOAc (v/v)); δ_H (500 MHz, D_6-Acetone): 9.30 (1H, d, *J* = 1.5 Hz, pyrazine 3-H), 8.62 (1H, dd, *J* = 2.5 & 1.5 Hz, pyrazine 6-H), 8.59 (1H, d, *J* = 2.5 Hz, pyrazine 5-H), 7.34 (1H, tt, *J* = 8.5 & 6.5 Hz, benzyl 4-H), 6.76 (2H, ap. t, *J* = 8.0 Hz), 4.63 (2H, s, benzyl CH_2); δ_c (125 MHz, D_6-Acetone): 169.3 (thiadiazole 5-C), 167.6 (thiadiazole 2-C), 163.2 (d, *J* = 7.0 Hz, benzyl 2-C), 161.3 (d, *J* = 7.0 Hz, benzyl 2-C), 147.3 (pyrazine 6-C), 145.7 (pyrazine 5-C), 145.4 (pyrazine 3-C), 142.6 (d, *J* = 3.5, benzyl 1-C), 131.6 (t, *J* = 9.5, benzyl 4-C), 112.6 (d, *J* = 5.5 Hz, benzyl 3-C), 112.5 (d, *J* = 5.5 Hz, benzyl 3-C), 26.3 (t, *J* = 3.5, benzyl-CH_2); δ_F (400 MHz, $CDCl_3$) -113.10 (ap. t, *J* = 6.5 Hz); ν_{max}/cm^{-1}; 3091.1, 3051.6, 2920.1, 2850.8, 1623.8, 1588.4, 1467.7; m/z ES+ Found MH^+ 323.0232 $C_{14}H_9F_2N_4S$ requires MH^+ 323.0231.

(pyrrolyl 3-C), 110.1 (pyrrolyl 4-C), 33.4 (benzyl CH_2); ν_{max}/cm^{-1}; 3174.6, 3133.4, 3105.4, 3074.9, 3002.2, 2938.0, 2851.7, 1614.6, 1473.0; m/z ES+ Found MH^+ 325.9920, $C_{13}H_{10}Cl_2N_3OS$ requires MH^+ 325.9916.

2-((2-chlorobenzyl)thio)-5-(pyrazin-2-yl)-1,3,4-thiadiazole (2b)

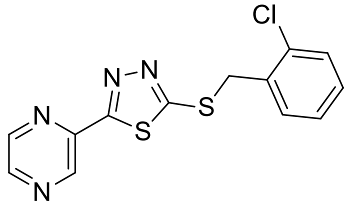

General produced A was followed using 5-(2-pyrazine)-1,3,4-thiadiazole-2-thiol (25 mg, 0.13 mmol), KOH (8 mg, 0.15 mmol) and 2-chlorobenzyl chloride (18 µL, 24 mg, 0.15 mmol) in DMF (2 mL) to afford a yellow solid (38 mg). This was purified by ACC (0-50% EtOAc in Petrol) to afford a yellow solid (28 mg, 0.09 mmol, 69%). Rf 0.55 (7:3 Petrol:EtOAc (v/v)); δ_H (500 MHz, $CDCl_3$): 9.55 (1H, bs, pyrazinyl 3-H), 8.67 (1H, d, *J* = 2.0 Hz, pyrazinyl 6-H), 8.60 (1H, bs, pyrazinyl 5-H), 7.65 (1H, dd, *J* = 7.0 & 2.0 Hz, benzyl 6-H), 7.43-7.42 (1H, m, benzyl 3-H), 7.29-7.24 (2H, m, pyrazinyl 4 & 5-H), 4.79 (2H, s, benzyl CH_2); δ_C (125 MHz, $CDCl_3$): 168.1 (thiadiazole 5-C), 167.8 (thiadiazole 2-C), 145.7 (pyrazinyl 6-C), 144.7 (pyrazinyl 2-C), 144.2 (pyrazinyl 5-C), 142.3 (pyrazinyl 3-C), 134.5 (benzyl C-1), 133.8 (benzyl 2-C), 131.5 (benzyl 6-C), 129.7 (benzyl 3-H), 129.5 (benzyl 5-C), 127.1 (benzyl 4-C), 35.7 (benzyl CH_2); ν_{max}/cm^{-1}; 3051.2, 2919.1, 2849.7, 1492.8, 1471.3, 1444.3.1402.8, 1347.92; m/z ES+ Found MH^+ 321.0026, $C_{13}H_9ClN_4S_2$ requires MH^+ 321.0030.

2-((3-chlorobenzyl)thio)-5-(pyrazin-2-yl)-1,3,4-thiadiazole (2c)

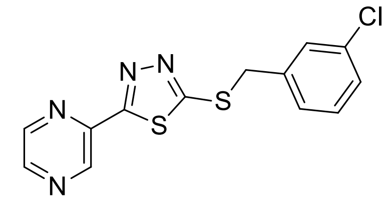

General produced A was followed using 5-(2-pyrazine)-1,3,4-thiadiazole-2-thiol (25 mg, 0.13 mmol), KOH (8 mg, 0.15 mmol) and 3-chlorobenzyl bromide (19 µL, 29 mg, 0.14 mmol) in DMF (2 mL) to afford an orange crystalline solid (64 mg). This was purified by ACC (0-50% EtOAc in Petrol) to afford a yellow solid (17 mg, 0.05 mmol, 38%). Rf 0.40 (7:3 Petrol:EtOAc (v/v)); δ_H (500 MHz, $CDCl_3$): 9.56 (1H, bs, pyrazinyl 3-H), 8.68 (1H, d, *J* = 2.0 Hz, pyrazinyl 6-H), 8.62 (1H, bs, pyrazinyl 5-H), 7.50 (1H, bs, benzyl 2-H), 7.40-7.39 (1H, m, benzyl 5-H), 7.31-7.30 (2H, m, benzyl 4 & 6-H), 4.64 (2H, s, benzyl CH_2); δ_C (100 MHz, $CDCl_3$): 167.8 (thiadiazole 5-C), 167.7 (thiadiazole 2-C), 145.8 (pyrazinyl 6-C), 144.7 (pyrazinyl 2-C), 144.2 (pyrazinyl 5-C), 142.3 (pyrazinyl 3-C), 137.9 (benzyl 1-C), 134.6 (benzyl 3-C), 130.0 (benzyl 4 or 6-C), 129.3 (benzyl 2-C), 128.2 (benzyl 4 or 6-C), 127.4 (benzyl 5-C), 37.3 (benzyl CH_2);ν_{max}/cm^{-1}; 3051.6, 2927.7, 2851.1,1520.9, 1495.9, 1475.0, 1429.9, 1402.8; m/z ES+ Found MH^+ 321.0026, $C_{13}H_9ClN_4S_2$ requires MH^+ 321.0030.

2-((4-chlorobenzyl)thio)-5-(pyrazin-2-yl)-1,3,4-thiadiazole (2d)

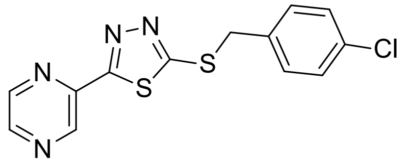

General produced A was followed using 5-(2-pyrazine)-1,3,4-thiadiazole-2-thiol (25 mg, 0.13 mmol), KOH (8 mg, 0.15 mmol) and 4-chlorobenzyl bromide (19 µL, 29 mg, 0.14 mmol) in DMF (2 mL) to afford a brown solid (31 mg, 0.10 mmol, 77%). Rf 0.40 (7:3 Petrol:EtOAc (v/v)); δ_H (500 MHz, $CDCl_3$): 9.56 (1H, bs, pyrazinyl 3-H), 8.68 (1H, d, *J* = 2.5 Hz, pyrazinyl 6-H), 8.62 (1H, bs, pyrazinyl 5-H), 7.45 (2H, d, *J* = 8.0 Hz, benzyl 2-H) 7.34 (2H, d, *J* = 8.0 Hz, benzyl 3-H), 4.63 (2H, s, benzyl CH_2); δ_C (100 MHz, $CDCl_3$): 167.8 (thiadiazole 5-C), 167.7 (thiadiazole 2-C), 145.7 (pyrazinyl 6-C), 144.7 (pyrazinyl 2-C), 144.2 (pyrazinyl 5-C), 142.3 (pyrazinyl 3-C), 134.4 (benzyl 1-C), 133.9 (benzyl 4-C), 130.6 (benzyl 2-C), 129.0 (benzyl 3-C), 37.3 (benzyl CH_2); ν_{max}/cm^{-1}; 3080.8, 3039.7, 2979.3, 2920.4, 2849.46, 1522.6, 1441.34, 1415.9, 1401.1; m/z ES+ Found MH^+ 321.0023, $C_{13}H_9ClN_4S_2$ requires MH^+ 321.0030.

2-((4-methoxybenzyl)thio)-5-(pyrazin-2-yl)-1,3,4-thiadiazole (2e)

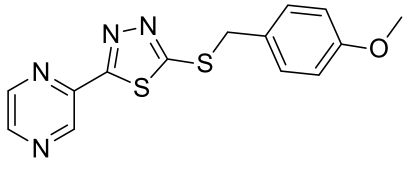

General produced A was followed using 5-(2-pyrazine)-1,3,4-thiadiazole-2-thiol (25 mg, 0.13 mmol), KOH (8 mg, 0.15 mmol) and 4-methoxybenzyl chloride (24 µL, 28 mg, 0.14 mmol) in DMF (2 mL) followed by trituration with Et_2O to afford a light brown solid (10 mg, 0.03 mmol, 23%). Rf 0.40 (7:3 Petrol:EtOAc (v/v)); δ_H (500 MHz, $CDCl_3$): 9.56 (1H, s, pyrazinyl 3-H), 8.68 (1H, bs, pyrazinyl 6-H), 8.62 (1H, bs, pyrazinyl 5-H), 7.41 (2H, d, *J* = 8.5 Hz, benzyl 2-H), 6.90 (2H, d, *J* = 8.5 Hz, benzyl 3-H), 4.62 (2H, s, benzyl CH_2), 3.83 (3H, s, OMe); δ_C (125 MHz, $CDCl_3$): 168.6 (thiadiazole 5-C), 167.4 (thiadiazole 2-C), 159.4 (benzyl 4-C), 145.58 (pyrazinyl 6-C), 144.9 (pyrazinyl 2-C), 144.2 (pyrazinyl

5-C), 142.3 (pyrazinyl 3-C), 130.5 (benzyl 2-C), 127.5 (benzyl 1-C), 114.5 (benzyl 3-C), 55.3 (OMe), 37.9 (benzyl CH_2); ν_{max}/cm^{-1}; 2916.1, 2838.6, 1606.9, 1580.6, 1510.4, 1493.6, 1401.9, 1341.8, 1298.5; m/z ES+ Found MH^+ 339.0341, $C_{14}H_{12}N_4OS_2$ requires MH^+ 339.0350.

2-((4-nitrobenzyl)thio)-5-(pyrazin-2-yl)-1,3,4-thiadiazole (2f)

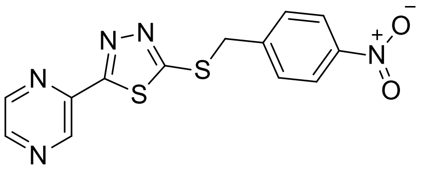

General produced B was followed using 5-(2-pyrazine)-1,3,4-thiadiazole-2-thiol (25 mg, 0.13 mmol), KOH (8 mg, 0.15 mmol) and 4-nitrobenzyl bromide (30 mg, 0.14 mmol) in DMF (2 mL) followed by trituration with Et_2O to afford an off-white powder (23 mg, 0.07 mmol, 54%). Rf 0.30 (7:3 Petrol:EtOAc (v/v)); δ_H (400 MHz, $CDCl_3$): 9.40 (1H, bs, pyrazinyl 3-H), 8.82 (1H, d, J = 2.5 Hz, pyrazonyl 6-H), 8.78 (1H, bs, pyrazinyl 5-H), 8.23 (2H, d, J = 8.5 Hz, benzyl 4-H), 7.80 (2H, d, J = 8.5 Hz, benzyl 3-H), 4.81 (2H, s, benzyl CH_2); δ_C (100 MHz, $CDCl_3$): 168.3 (thiadiazole 5-C), 167.8 (thiadiazole 2-C), 147.4 (pyrazinyl 2-C), 147.1 (pyrazinyl 6-C), 145.4 (pyrazinyl 5-C), 145.3 (benzyl 4-C), 144.2 (benzyl 1-C), 141.9 (pyrazinyl 3-C), 130.9 (benzyl 2-C), 124.2 (benzyl 3-C), 36.9 (benzyl CH_2); ν_{max}/cm^{-1}; 3083.2, 2982.6, 29276, 2843.4, 1595.6, 1509.1, 1491.1, 1416.7 1405.2, 1394.1, 1353.7, 1341.7, 1307.2; m/z ES+ Found MNa^+ 354.0085, $C_{13}H_9N_5O_2S_2$ requires MNa^+ 353.0090.

2-((2-chloro-6-fluorobenzyl)thio)-5-(pyrazin-2-yl)-1,3,4-thiadiazole (2g)

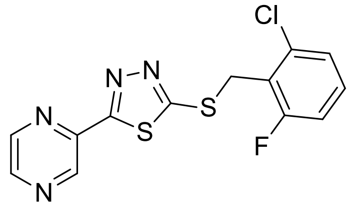

General produced A was followed using 5-(2-pyrazine)-1,3,4-thiadiazole-2-thiol (25 mg, 0.13 mmol), KOH (8 mg, 0.15 mmol) and 2-chloro-6-fluorobenzyl chloride (18 µL, 25 mg, 0.14 mmol) in DMF (2 mL) to afford a white crystalline solid (14 mg, 0.04 mmol, 31%). Rf 0.45 (7:3 Petrol:EtOAc (v/v)); δ_H (400 MHz, d_6-Acetone): 9.31 (1H, d, J = 1.5 Hz, pyrazinyl 3-H), 8.65 (1H, d, J = 2.5 Hz, pyrazinyl 6-H, 8.61-8.60 (1H, m, pyrazinyl 5-H), 7.36-7.30 (1H, m benzyl 4-H) 7.25 (1H, d, J = 8.0 Hz, benzyl 3-H), 7.12-7.08 (1H, m, benzyl 5-H), 4.74 (2H, d, J = 1.5 Hz, Benzyl CH_2) δ_C (100 MHz, $CDCl_3$): 168.0 (thiadiazole 5-C), 167.7 (thiadiazole 2-C), 162.8 (benzyl 6-C), 160.3 (benzyl 2-C), 145.8 (pyrazinyl 6-C), 144.8 (pyrazinyl 2-C), 1442 (pyrazinyl 5-C), 142.4 (pyrazinyl 3-C), 130.1 (benzyl 4-C), 125.6 (benzyl 3-C), 122.0 (benzyl 1-C), 114.4 (benzyl 5-C), 29.6 (benzyl CH_2); ν_{max}/cm^{-1}; 3085.9. 2956.9, 2920.4, 2851.1, 1604.6, 1576.7, 1460.0, 1449.4, 1424.7, 1402.1, 1354.0; m/z ES+ Found MH^+ 338.9890, $C_{13}H_8ClFN_4S_2$ requires MH^+ 338.9941.

2-(benzylthio)-5-(pyrazin-2-yl)-1,3,4-thiadiazole (2h)

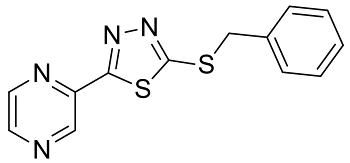

General produced A was followed using 5-(2-pyrazine)-1,3,4-thiadiazole-2-thiol (25 mg, 0.13 mmol), KOH (8 mg, 0.15 mmol) and benzyl chloride (17 µL, 24 mg, 0.14 mmol) in DMF (2 mL) to afford a brown crystalline solid (30 mg, 0.10 mmol, 77%). Rf 0.50 (7:3 Petrol:EtOAc (v/v)); δ_H (400 MHz, $CDCl_3$): 9.45 (1H, d, *J* = 1.5 Hz, pyrazinyl 3-H), 8.57 (1H, d, *J* = 2.5 Hz, pyrazinyl 6-H), 8.51-8.50 (1H, m, pyrazinyl 5-H), 7.49 (2H, d, *J* = 7.0 Hz, benzyl 2-H), 7.30-7.19 (3H, m, benzyl 3 & 4-H), 4.57 (2H, s, benzyl CH_2); δ_C (100 MHz, $CDCl_3$): 168.3 (thiadiazole 5-C), 167.6 (thiadiazole 2-C), 145.7 (pyrazinyl 6-C), 144.8 (pyrazinyl 2-C), 144.2 (pyrazinyl 5-C), 142.3 (pyrazinyl 3-C), 135.6 (benzyl 1-C), 129.2 (benzyl 2-C), 128.8 (benzyl 3-C), 128.0 (benzyl 4-C), 38.2 (benzyl CH_2); ν_{max}/cm^{-1}; 3082.3, 3051.0, 2978.7, 2926.8, 2849.3, 1494.6, 1455.6, 1403.6, 1350.3, 1310.2; m/z ES+ Found MH^+ 287.0370, $C_{13}H_{11}N_4S_2$ requires MH^+ 287.0425.

2-{[(2,6-dichlorophenyl)methyl]sulfanyl}-5-phenyl-1,3,4-thiadiazole (2i)

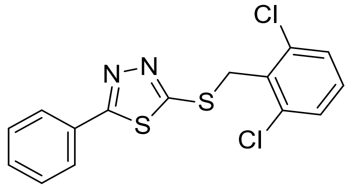

General procedure A was followed using 5-phenyl-1,3,4-thiadiazole-2-thiol (50 mg, 0.26 mmol), KOH (17 mg, 0.31 mmol) and 2,6-dichlorobenzyl chloride (57 mg, 0.29 mmol) to afford an off white powder with no further purification necessary (53 mg, 0.15 mmol, 58%) R_f 0.80 (7:3 EtOAc:Petrol (v/v));; δ_H (500 MHz, $CDCl_3$): 7.83 (2H, dd, *J* = 7.8 Hz & *J* = 1.7 Hz, phenyl 2-H), 7.41 (3H, m, phenyl 3,4-H), 7.27 (2H, d, *J* = 8.0 Hz, dichlorophenyl 3-H), 7.13 (1H, t, *J* = 8.0 Hz, dichlorophenyl 4-H), 4.39 (1H, s, CH_2-S); δ_C (125 MHz, $CDCl_3$) 169.2 (thiadiazole 2-C) 164.1 (thiadiazole 5-C), 136.3 (dichlorophenyl 2-C), 131.9 (phenyl 1-C), 131.2 (phenyl 4-C), 129.9 (phenyl 1-C) 129.8 (dichlorophenyl 5-C), 129.2 (phenyl 3-C) 128.5 (dichlorophenyl 3-C), 127.8 (phenyl 2-C), 34.7(methylene); ν_{max}/cm^{-1}; 3076, 3008, 2922, 2852, 1578, 1559; m/z ES+ Found MH^+, 352.9742 $C_{15}H_{10}Cl_2N_2OS_2$ requires MH^+, 352.9735.

2-(5-{[(2,6-dichlorophenyl)methyl]sulfanyl}-1,3,4-oxadiazol-2-yl)pyridine (2j)

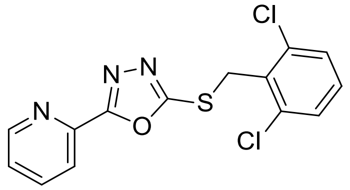

General procedure A was followed using 5-(2-pyridinyl)-1,3,4-oxadiazole-2-thiol (50 mg, 0.28 mmol), KOH (19 mg, 0.34 mmol) and 2,6-dichlorobenzyl chloride (61 mg, 0.31 mmol) to afford a white powder (70 mg, 0.21 mmol, 75%) R_f 0.40 (7:3 EtOAc:Petrol (v/v)); δ_H (500 MHz, $CDCl_3$): 8.80 (1H, d, *J* = 4.2 Hz, pyridine 6-H), 8.24 (1H, d, *J* = 7.9 Hz, pyridine 3-H), 7.92 (1H, t, *J* = 7.9 Hz, pyridine 5-H), 7.49 (1H, dd, *J* = 7.1 Hz & *J* = 5.2 Hz, pyridine 4-H), 7.37 (2H, d, *J* = 8.1 Hz, phenyl 3-H), 7.24 (1H, t, , *J* = 8.1 Hz, phenyl 4-H), 4.93 (2H, s, benzyl CH_2); δ_C (125 MHz, $CDCl_3$) 165.2 (oxadiazole 2-C), 165.1 (oxadiazole 5-C), 150.3 (pyridine 6-C), 143.2 (pyridine 2-C), 137.3 (pyridine 5-C), 136.3 (pheny 2-C),

131.4 (phenyl 1-C), 130.0 (phenyl 4-C), 128.6 (phenyl 3-C), 125.9 (pyridine 4-C), 122.9 (pyridine 3-C), 33.2 (methylene); ν_{max}/cm^{-1}; 3074, 2922, 2852, 1589, 1579, 1558; m/z ES+ Found MH^+, 337.9927. $C_{14}H_{10}Cl_2N_3OS$ requires MH^+, 337.9916.

2-((2,6-dichlorobenzyl)thio)-5-(1H-pyrrol-2-yl)-1,3,4-oxadiazole (2k)

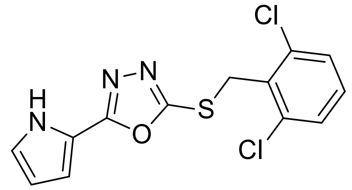

General procedure A was followed using 5-(1H-pyrol-2-yl)-1,3,4-oxadiazole-2-thiol (50 mg, 0.30 mmol), KOH (20 mg, 0.33 mmol) and 2,6-dichlorochlorobenzyl chloride (59 mg, 0.30 mmol) to afford a fluffy white solid (77 mg, 0.24 mmol, 80%). Rf 0.5 (7:3 Petrol:EtOAc (v/v)); δ_H (500 MHz, d_6-DMSO): 12.20 (1H, br s, pyrrol 1-H), 7.52 (2H, d, J = 8 Hz, benzyl 3-H), 7.40 (1H, dd, J = 8.5 & 7.5, benzyl 4-H), 7.08 (1H, m, pyrrole 5-H), 6.77 (1H, m, pyrrolyl 3-H), 6.26 (1H, m, pyrrolyl 4-H), 4.67 (2H, s, benzyl CH_2); δ_C (125 MHz, d_6-DMSO): 161.2 (oxadiazole 5-C), 159.3 (oxadiazole 2-C), 134.9 (benzyl 2-C), 132.0 (benzyl 1-C), 130.7 (benzyl 4-C), 128.8 (benzyl 3-C), 124.0 (pyrrolyl 5-C), 114.8 (pyrrolyl 2-C), 112.4

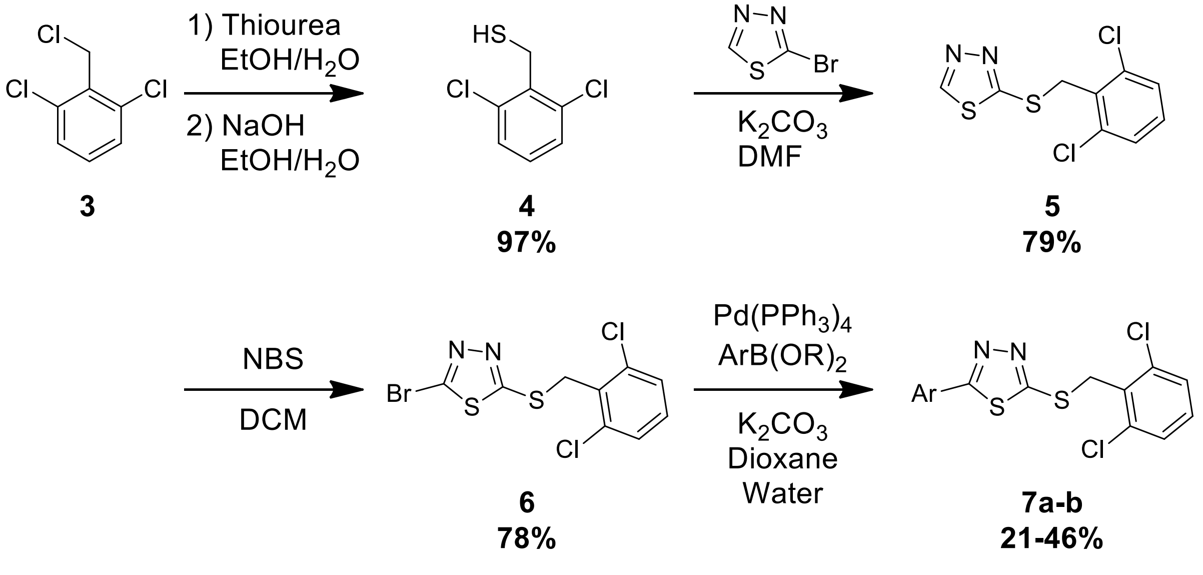

*Figure S2: General synthetic route towards **7a** and **7b**. 2,6-dichlorobenzyl chloride (**3**) is first converted to the thiol **4** followed by an S_NAr to give **5**, which is then brominated to give **6** ready for a Suzuki cross-coupling to give the desired products **7a-b**.*

2,6,-dichlorobenzyl thiol (4)

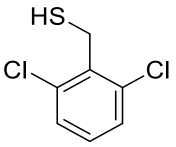

2,6,-dichlorobenzyl chloride (3.00 g 16.4 mmol) and thiourea (1.38 g, 18.4 mmol) were dissolved in EtOH (36 mL) and H_2O (14 mL) then heated to reflux for 3 h. The solution was cooled to room temperature and dissolved in 2 M aq. NaOH (30 mL) and EtOH (10 mL) and the reaction was refluxed for a further 3 hours. The solution was then cooled, reduced *in vacuo*, taken up in water (30 mL), acidified and extracted with EtOAc (3 x 20 mL). The combined organic layers were then washed with sat. $NaHCO_3$ solution (2 x 15 mL), sat. NaCl solution (2 x 20 mL), dried over Na_2SO_4 and evaporated to dryness to afford an off white crystalline solid (206 mg). This was purified by ACC (0-100% EtOAc in Petrol) to afford an off white crystalline solid (2.90 g, 15.0 mmol, 97%). Rf 0.7 (9:1 Petrol:EtOAc (v/v)); δ_H (500 MHz, $CDCl_3$): 7.33 (2H, d, J = 8.0 Hz, benzyl 3-H), 7.15 (1H, t, J = 8.0 Hz, benzyl 4-H), 4.02 (2H, d, J = 8.5 Hz, benzyl CH_2), 2.12 (1H, t, J = 8.5 Hz, SH); δ_C (125 MHz, $CDCl_3$): 137.3 (benzyl 1-C), 134.6 (benzyl 2-C), 128.5 (benzyl 4-C), 128.5 (benzyl 3-C), 24.4 (benzyl CH_2); ν_{max}/cm^{-1}; 3370.7, 2925.4, 1603.3, 1579.3, 1560.8, 1521.7.

2-((2,6-dichlorobenzyl)thio)-1,3,4-thiadiazole (5)

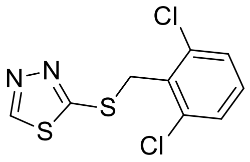

2,6-dichlorobenzyl thiol (2.85 g, 14.77 mmol), 2-bromo-1,3,4-thiadiazole (2.43 g, 14.77 mmol),& K_2CO_3 (2.38 g, 17.72 mmol) were dissolved in DMF (10 mL) and heated to 90 °C for 18 h. The reaction was diluted with H_2O (100 mL), extracted with EtOAc (3 x 40 mL) and the organic layers combined. These were washed with brine (3 x 40 mL), 10% LiCl (3 x 40 mL), dried over $MgSO_4$, filtered and reduced *in vacuo* to give brown residue (4.33 g). This was purified by ACC (0-30% EtOAc in Petrol) to afford white crystalline solid (2.33 g, 8.43 mmol, 57 %) Rf 0.60 (7:3 Petrol:EtOAc (v/v)); δ_H (400 MHz, $CDCl_3$): 8.99 (1H, s, thiadiazole 5-H), 7.27 (2H, d, J = 8.5 Hz, benzyl 3-H), 7.13 (1H, t, J = 8.5 H, benzyl 4-H), 4.89 (2H, s, benzyl CH_2); δ_C (100 MHz, $CDCl_3$): 164.7 (thiadiazole 2-C), 152.1 (thiadiazole 5-C), 136.3 (benzyl 2-C), 131.7 (benzyl 1-C), 129.8 (benzyl 4-C), 128.5 (benzyl 3-C), 34.73 (benzyl CH_2); ν_{max}/cm^{-1}; 1580.5, 1560.4, 1434.7, 1410.9, 1365.2; m/z ES+ Found MNa^+ 298.9235, $C_9H_6Cl_2N_2S_2$ requires MNa^+ 298.9242.

2-bromo-5-((2,6-dichlorobenzyl)thio)-1,3,4-thiadiazole (6)

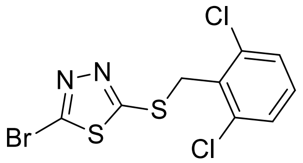

2-((2,6-dichlorobenzyl)thio)-1,3,4-thiadiazole (2.33 g, 8.43 mmol), & N-bromosuccinimide (2.10 g, 11.80 mmol) were dissolved in DCM (10 mL) and refluxed for 48 h. The reaction was then cooled, quenched with sat. $Na_2S_2O_3$ (20 mL), partitioned and the aqueous extracted with DCM (2 x 15 mL). The organic layers were combined, dried over $MgSO_4$, filtered an reduced *in vacuo* to give an orange, oily crystals (3.15 g). This was purified by ACC (0-30% EtOAc in Petrol) to afford a white crystalline solid (2.80 g, 7.87 mmol, 78 %) Rf 0.80 (7:3 Petrol:EtOAc (v/v)); δ_H (400 MHz, $CDCl_3$): 7.37 (2H, d,

J = 8 Hz, benzyl 3-H), 7.24 (1H, dd, , J = 8.5 & 7.5 Hz, benzyl 4-H), 4.92 (2H, s benzyl CH_2); δ_C (100 MHz, $CDCl_3$): 168.1 (thiadiazole 5-C), 138.1 (thiadiazole 2-C), 136.3 (benzyl 2-C), 131.5 (benzyl 1-C), 129.9 (benzyl 4-C), 128.6 (benzyl 3-C), 34.6 (benzyl CH_2); ν_{max}/cm^{-1}; 3068.4, 2956.7, 1576.5, 1558.4, 1434.3, 1422.6, 1405.1; m/z ES+ Found MH^+ 356.8462, $C_9H_5BrCl_2N_4S_2$ requires MH^+ 356.8512.

General Procedure B

The desired aromatic halide (0.28 mmol, 1 eq), the desired boronic acid/ester (0.28 mmol, 1 eq) and K_2CO_3 (1.12 mmol, 4 eq) were dissolved in dioxane (2 mL) and H_2O (2 mL) then degassed with N_2 for 30 minutes. $Pd(PPh_3)_4$ (0.04 mmol, 0.15 eq) was then added and the reaction was then heated to 90 °C for 2-6 h. Upon completion, the reaction was diluted with H_2O (20 mL), extracted with DCM (3 x 10 mL), dried over Na_2SO_3, filtered and reduced *in vacuo* to afford the crude product.

2-((2,6-dichlorobenzyl)thio)-5-(pyridin-3-yl)-1,3,4-thiadiazole (7a)

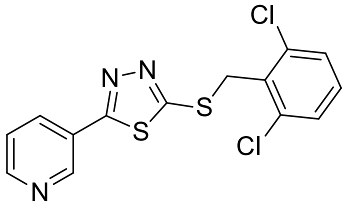

General procedure B was following using 2-bromo-5-((2,6-dichlorobenzyl)thio)-1,3,4-thiadiazole (50 mg, 0.14 mmol), 3-pyridine boronic acid pinacol ester (29 mg, 0.14 mmol), $Pd(PPh_3)_4$ (23 mg, 0.02 mmol), K_2CO_3 (77 mg, 0.56 mmol), dioxane (1 mL) and water (1 mL) to afford crude brown solid (74 mg). This was purified by ACC (0-60% EtOAc in Petrol) and the resultant product dissolved in EtOAc (5 mL) with QuadraSil to remove residual metals to afford a pale brown solid (21 mg, 0.06 mmol, 43%). Rf 0.30 (7:3 Petrol:EtOAc (v/v)); δ_H (400 MHz, $CDCl_3$): 9.01 (1H, bs, pyridinyl 2-H), 8.65 (1H, d, J = 5.0 Hz, pyridinyl 4-H), 8.20 (1H, ap. dt, J = 8 & 1.5 Hz, pyridinyl 6-H), 7.37 (1H, dd, J = 8.0 & 5.0 Hz, pyridinyl 5-H), 7.28 (2H, d, J = 8.0 Hz, benzyl 3-H), 7.14, t, J = 8.0 Hz, benzyl 4-H), 4.92 (2H, s, benzyl CH_2); δ_C (100 MHz, $CDCl_3$): 165.7 (thiadiazole 5-C), 165.1 (thiadiazole 2-C), 151.9 (pyridinyl 4-C), 148.7 (pyridinyl 2-C), 136.3 (benzyl 2-C), 134.6 (pyridinyl 6-C), 131.7 (benzyl 1-C), 129.9 (pyridinyl 5-C), 128.6 (benzyl 3-C), 126.3 (pyridinyl 3-C), 124.0 (benzyl 4-C), 34.6 (benzyl CH_2); ν_{max}/cm^{-1}; 3061.4, 3003.1, 2953.6, 2917.4, 2850.9, 1580.8, 1561.0, 1461.4, 1434.7 1407.3, 1362.8, 1336.6, 1270.2; m/z ES+ Found MH^+ 353.9649, $C_{14}H_9Cl_2N_3S_2$ requires MH^+ 353.9693.

2-((2,6-dichlorobenzyl)thio)-5-(1H-pyrrol-2-yl)-1,3,4-thiadiazole (7b)

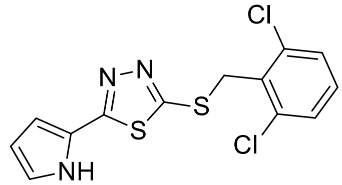

General procedure B was followed using 2-bromo-5-((2,6-dichlorobenzyl)thio)-1,3,4-thiadiazole (100 mg, 0.28 mmol), N-(Boc)-pyrrol-2-boronic acid (60 mg, 0.28 mmol), K_2CO_3 (155 mg, 1.12 mmol), $Pd(PPh_3)_4$ (46 mg, 0.04 mmol), dioxane (2mL) and water (2 mL) to afford crude yellow solid (78 mg). This was triturated with DCM (2 mL) to afford the desired product as a white crystalline solid (26 mg, 0.08 mmol, 29%) Rf 0.55 (7:3 Petrol:EtOAc (v/v)); δ_H (400 MHz, $CDCl_3$): 12.10 (1H, bs, pyrrolyl N-H), 7.54 (2H, d, J = 8.0 Hz, benzyl 3-H), 7.41 (1H, t, 8.0 Hz, benzyl 4-H), 7.04 (1H, bs, pyrrolyl 5-H),

6.75 (1H, d, J = 3.0 Hz, pyrrolyl 3-H), 6.23 (1H, ap. t, J = 2.7 Hz, pyrrolyl 4-H), 4.74 (2H, s, benzyl CH_2); δ_C (100 MHz, $CDCl_3$): 162.8 (thiadiazole 2-C), 159.4 (thiadiazole 5-C), 135.6 (benzyl 2-C), 132.4 (benzyl 1-C), 131.2 (benzyl 4-C), 129.3 (benzyl 3-C), 123.8 (pyrrolyl 5-C), 121.6 (pyrrolyl 2-C), 113.8 (pyrrolyl 3-C), 110.7 (pyrrolyl 4-C), 35.5 (benzyl CH_2); ν_{max}/cm^{-1}; 3185.1, 314.7, 3078.5, 3008.5, 1570.4, 1559.8, 1471.3, 1433.1, 1386.2; m/z ES+ Found MH^+ 341.9652, $C_{13}H_9Cl_2N_3S_2$ requires MH^+ 341.9693.

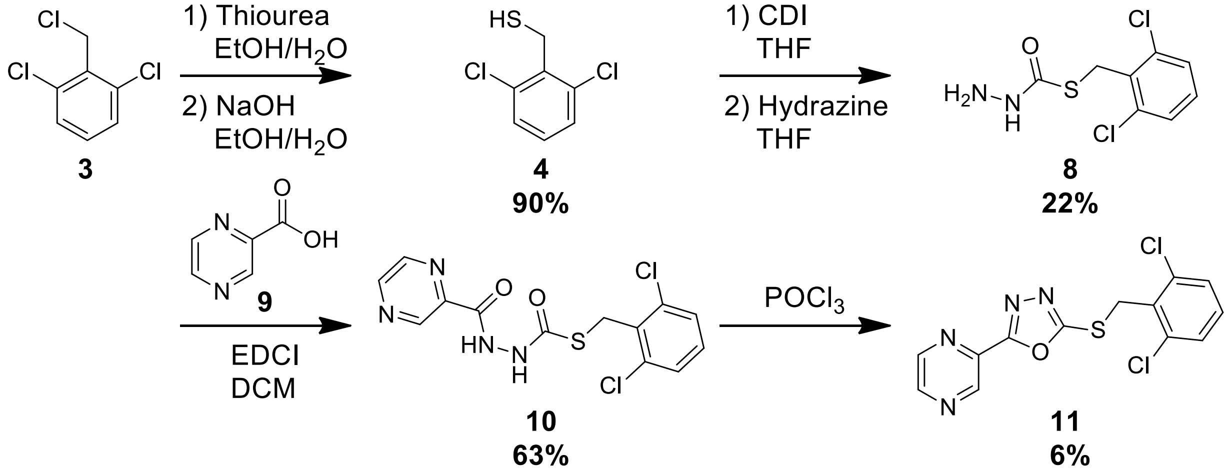

Figure S3: Synthetic route for ***10****. 2,6-dichlorobenzyl chloride (3) is first converted to the thiol* ***4*** *ready for a reaction with CDI and hydrazine to afford* ***8****. Compound* ***8*** *is then utilised in an amide coupling with* ***9*** *using EDCI to produce* ***10*** *ready for a cyclising-condensation reaction to afford* ***11****.*

***S*-2,6-dichlorobenzyl hydrazinecarbothioate (8)**

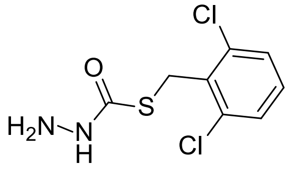

2,6-dichlorobenzyl thiol (872 mg, 4.52 mmol) & CDI (806 mg, 4.97 mmol) were dissolved in THF (10 mL) and left to stir under N_2 for 24 h before adding hydrazine monohydrate (263 µL, 5.42 mmol) and leaving to stir for a further 48 h. The solvent was removed *in vacuo* and the residues take up in H_2O (40 mL) and DCM (40 mL), partitioned, the aqueous extracted with DCM (2 x 30 mL), organic combined and washed with sat. NaCl solution (3 x 30 mL), dried over $MgSO_4$, filtered and the solvent removed *in vacuo* to afford a white residue (869 mg), This was purified by ACC (0-100% EtOAc in petrol) to afford a white residue (306 mg, 1.22 mmol, 27 %).δ_H (400 MHz, $CDCl_3$): 7.42 (1/2 H, bs, hydrazinyl 1-H), 7.30 (2H, d, J = 8.0 Hz, benzyl 3-H), 7.14 (1H, t, J = 8.0 Hz, benzyl 4-H), 7.06 (1/2 H, bs, hydrazinyl 1-H), 4.50 (4.50 2H, bs, benzyl CH_2), 3.88 (1H, bs, hydrazinyl 2-H), 3.77 (1H, bs, hydrazinyl 2-H); δ_C (100 MHz, $CDCl_3$): 136.0 (benzyl 2-C), 133.2 (benzyl 1-C), 129.1 (benzyl 4-C), 128.4 (benzyl 3-C), 29.9 (benzyl CH_2); LC-MS m/z ES+ Found MH^+ 251.0.

S-2,6-dichlorobenzyl 2-(pyrazine-2-carbonyl)hydrazinecarbothioate (10)

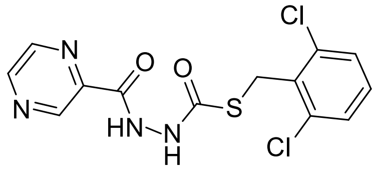

S-2,6-dichlorobenzyl hydrazinecarbothioate (250 mg, 1.00 mmol) and EDCI (210 mg 1.10 mmol), were dissolved in DCM (10 Ml) and stirred for 20 mins. 2-pyrazine carboxylic acid (223 mg, 1.8 mmol) was added and the solution was left to stir for 18 h. The solvent was then removed *in vacuo,* the remaining residue dissolved in EtOAc (20 mL) and 2 M HCl (2 mL) and partitioned. The aqueous layer was extracted with EtOAc, (2 x 20 mL), the organic layers combined and then washed with sat. $NaHCO_3$ (3 x 30 mL), sat NaCl (3 x 20 mL), dried over $MgSO_4$, filtered and solvent removed to afford a pale yellow solid (226 mg, 0.63 mmol, 63%). δ_H (400 MHz, $CDCl_3$): 9.42 (1H, d, *J* = 1.5 Hz, pyrazinyl 3-H), 8.84 (1H, d, *J* = 2.5 Hz, pyrazinyl 6-H), 8.58-8.57 (1H, m, pyrazinyl 5-H), 7.32 (2H, d, *J* = 8.0 Hz, benzyl 3-C), 7.17 (1H, t, *J* = 8.0 Hz, benzyl 4-H), 4.60 (2H, s, benzyl CH_2); m/z ES+ Found MNa^+ 378.9765, $C_{13}H_{10}Cl_2N_4O_2S$ requires MNa^+ 379.9799.

2-((2,6-dichlorobenzyl)thio)-5-(pyrazin-2-yl)-1,3,4-oxadiazole (11)

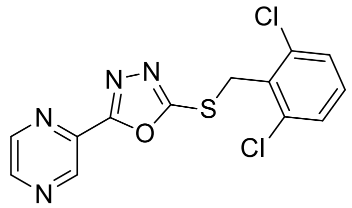

S-2,6-dichlorobenzyl 2-(pyrazine-2-carbonyl)hydrazinecarbothioate (100 mg, 0.27 mmol) was dissolved in $POCl_3$ (0.4 mL) and refluxed for 2 h under N_2. The reaction was cool and poored into a flask of 0 °C water (20 mL). This was neutralised using sat. NH_4OH (20 mL), extracted with DCM (3 x 20 mL), dried of $MgSO_4$, filtered and reduced *in vacuo* to afford a crude oil (54 mg). This was purified by ACC (0-10% EtOAc/Petrol) to afford a colourless solid (5 mg, 0.02 mmol, 6 %). Rf 0.45 (7:3 Petrol:EtOAc (v/v)); δ_H (400 MHz, $CDCl_3$): 9.47 (1H, bs, pyrazinyl 3-H), 8.76 (2H, bs, pyrazinyl 5 & 6-H), 7.38 (2H, d, *J* = 8 Hz, benzyl 3-H), 7.25 (1H, t, *J* = 8 Hz, benzyl 4-H), 4.95 (2H, s, benzyl CH_2); δ_C (100 MHz, $CDCl_3$): 165.9 (oxadiazole 5-C), 163.4 (oxadiazole 2-C), 146.5 (pyrazinyl 5 or 6-C), 144.6 (pyrazinyl 5 or 6-C), 144.0 (pyrazinyl 3-C), 139.4 (pyrazinyl 2-C), 136.3 (benzyl 2-C), 131.2 (benzyl 1-C), 130.1 (benzyl 3-C), 128.6 (benzyl 4-C), 33.2 (benzyl CH_2); ν_{max}/cm^{-1}; 3067.3, 3011.9, 2917.9, 2850.7, 1579.7, 1560.5, 1456.2, 1439.7, 1413.9; m/z ES+ Found MH^+ 338.9833, $C_{13}H_8Cl_2N_4OS$ requires MH^+ 388.9874.
